# The roles of the *Listeria monocytogenes* post-translocation chaperones PrsA1 and PrsA2 in protein secretion and stress resistance

**DOI:** 10.1128/jb.00531-25

**Published:** 2025-12-29

**Authors:** Jada L. George, Leah F. Cabo, Jon P. Boyle, Nancy E. Freitag, Laty A. Cahoon

**Affiliations:** 1 Department of Biological Sciences, University of Pittsburgh 6614, Pittsburgh, Pennsylvania, USA; 2 Department of Microbiology and Immunology, University of Illinois at Chicago 21725, Chicago, Illinois, USA; 3 Department of Pharmaceutical Sciences, University of Illinois Chicago 14681, Chicago, Illinois, USA; University of Illinois Chicago, Chicago, Illinois, USA

**Keywords:** gram-positive secretion, *Listeria monocytogenes*, PPIase, parvulin, chaperone, PrsA1, PrsA2, stress resistance, secretome

## Abstract

PrsA1 and PrsA2 are parvulin peptidyl-prolyl isomerases that function as post-translocation secretion chaperones in *Listeria monocytogenes*. To assess the contributions of PrsA1 and PrsA2 to overall *L. monocytogenes* protein secretion, we analyzed *prsA1* and *prsA2* deletion mutants and PrsA2 structural variants for altered secretion profiles when compared to wild-type bacteria using tandem mass-tagged mass spectrometry. We find that *prsA1* and *prsA2* deletion mutants have distinctly altered secretion profiles. In addition, among the subset of known secreted proteins with significantly altered secretion abundance were those with characterized functions in virulence, cell division and cell wall assembly, and stress response. To further identify common pathways and protein factors that are altered when PrsA homologs are absent, we conducted a meta-analysis comparing our data from *L. monocytogenes* to recently published quantitative proteomic secretome data of *prsA* deletion mutants from diverse gram-positive human pathogens, including *Streptococcus pneumoniae*, *Streptococcus pyogenes*, and *Staphylococcus aureus*. We find that when PrsA homologs are absent in diverse gram-positive bacteria, several pathways are similarly affected, including those promoting bacterial virulence, cell division and cell wall assembly, and oxidative stress resistance. Moreover, we provide evidence of novel roles for *L. monocytogenes* PrsA1 and PrsA2 in oxidative stress resistance and cell morphology, and PrsA2 in thermo-osmotic stress resistance. Overall, this work suggests that gram-positive PrsA homologs serve in the maturation of multiple protein substrates with varied cellular functions.

**IMPORTANCE:**

Bacterial protein secretion is critical for functions ranging from cell physiology to virulence. Here, we examine the effect of deleting two *Listeria monocytogenes* secretion chaperones, PrsA1 and PrsA2, and find that in the absence of one or both chaperones, secretion of several proteins implicated in key biological processes was significantly disrupted. These results, coupled with phenotypic observations of chaperone deletion mutants, reveal that PrsA1 and PrsA2 have roles in bacterial physiology and stress resistance. Furthermore, our meta-analysis of prsA deletion mutants in *Streptococcus pneumoniae*, *Streptococcus pyogenes,* and *Staphylococcus aureus* suggests that the contribution of PrsA to critical bacterial processes is well conserved in gram-positive pathogens. Our work lays the foundation for future inquiry investigating the client repertoire of these chaperones.

## INTRODUCTION

*Listeria monocytogenes* is a gram-positive bacterium that typically exists as a saprophyte in the soil and on decaying plant matter but can also transition into a facultative intracellular pathogen capable of infecting mammalian hosts (1–4). *L. monocytogenes* can survive under a wide range of environmental conditions, including temperatures ranging from 0.4°C to 45°C, salt concentrations up to 10%, and extreme acidic and basic environments with pH ranging from 4.4 to 10 (2–4). Infection with *L. monocytogenes* typically occurs through the consumption of contaminated foods such as dairy, produce, and processed meats and typically causes mild forms of disease in healthy individuals (5, 6). However, in susceptible populations such as the elderly, children, pregnant women, and the immunocompromised, the bacterium can cause listeriosis, which is linked to illnesses such as sepsis, encephalitis, and meningitis (5, 6). In pregnant women, *L. monocytogenes* can cross the placenta and infect the developing fetus, leading to stillbirth and abortion (6, 7). The capability of *L. monocytogenes* to survive in diverse and challenging environments outside of the human host has resulted in the bacterium becoming a frequent contaminant of food processing facilities, resulting in some of the largest and most deadly food recalls in the United States (8–10), and accounting for ~45% of the biological contamination food recalls in the United States between 2002 and 2023 (11).

The maturation of secreted proteins is critical for the growth and survival of bacterial cells. In gram-positive bacteria such as *L. monocytogenes*, after post-translocation across the bacterial cell membrane, secreted proteins must fold and become active in the space between the cell membrane and the thick peptidoglycan cell wall. Due to the presence of teichoic and lipoteichoic acids, which span the peptidoglycan, the membrane-wall space is filled with a high density of negative charge. In addition, this space is solvent-exposed, which, in combination with negative charge, hampers the ability of many secreted proteins to self-fold, thereby necessitating the presence of secreted chaperones (12).

In the membrane-cell wall space of *L. monocytogenes,* peptidyl-prolyl isomerases (PPIases), PrsA1 and PrsA2, function as post-translocation secretion chaperones assisting in the secretion and folding of proteins with diverse functions (13). Specifically, they share homology with the parvulin family of PPIases, which catalyze the *cis*-to-*trans* conversion of peptides N-terminal to proline residues (14, 15). PrsA1 and PrsA2 are dimeric proteins, formed by an N-terminal domain swap of two monomers, which allows for the formation of a foldase pocket critical for chaperone activity (16, 17). Additionally, a lipid-modified N-terminal cysteine tethers these secretion chaperones to the bacterial cell membrane (18). PrsA1 and PrsA2 share 58% sequence identity and 75% sequence similarity yet demonstrate a divergence in their functions associated with the bacterial cell (19). Deletion of PrsA1 results in decreased *L. monocytogenes* resistance to ethanol stress and reduced bacterial translocation across the small intestine in an intra-gastric mouse model of infection (16, 20). In contrast, PrsA2-deficient *L. monocytogenes* exhibit significant reductions in virulence in both the septicemic and intra-gastric mouse infection models, in addition to reductions in swimming motility, resistance to acidic and basic pH stress, and growth under high osmolarity (16, 19–21). Because of their sequence similarity and divergent activities, the structures of these chaperones have been investigated, and key amino acids have been identified in PrsA1 and PrsA2, which confer membrane localization, dimerization, and foldase domain function (16).

Thus far, investigations into the contributions of PrsA2 to the *L. monocytogenes* secretome have been limited in scope and somewhat limited in sensitivity, with approaches focusing mostly on virulence factor secretion (17, 22). Given that secreted proteins are critical for *L. monocytogenes* physiology and the bacterium is capable of surviving in many diverse environments, including within mammalian host cells and tissues, we sought to characterize the full spectrum of proteins that rely on PrsA1 and PrsA2 for proper secretion. In this work, we use tandem mass-tagged mass spectrometry (TMT-MS), a sensitive method to analyze secreted protein levels, in combination with stress resistance assays and microscopy to further elucidate the functions of PrsA1 and PrsA2 in *L. monocytogenes*. We have further conducted a meta-analysis to compare our TMT-MS secretome data from *L. monocytogenes* to published secretome data from *prsA* homolog deletion mutants from diverse gram-positive bacteria, including *Streptococcus pneumoniae*, *Streptococcus pyogenes*, and *Staphylococcus aureus* (23–25). We find that there are multiple shared pathways dependent on PrsA in each of these gram-positive human pathogens, and these pathways include those promoting bacterial virulence, cell division and cell wall assembly, and oxidative stress resistance. Our findings emphasize the broad and often functionally conserved roles that PrsA homologs serve in the maturation of secreted protein factors across gram-positive species.

## RESULTS

### In-depth proteomic analysis reveals altered secretion profiles for *L. monocytogenes* PrsA1 and PrsA2 mutants

**Fig 1:**
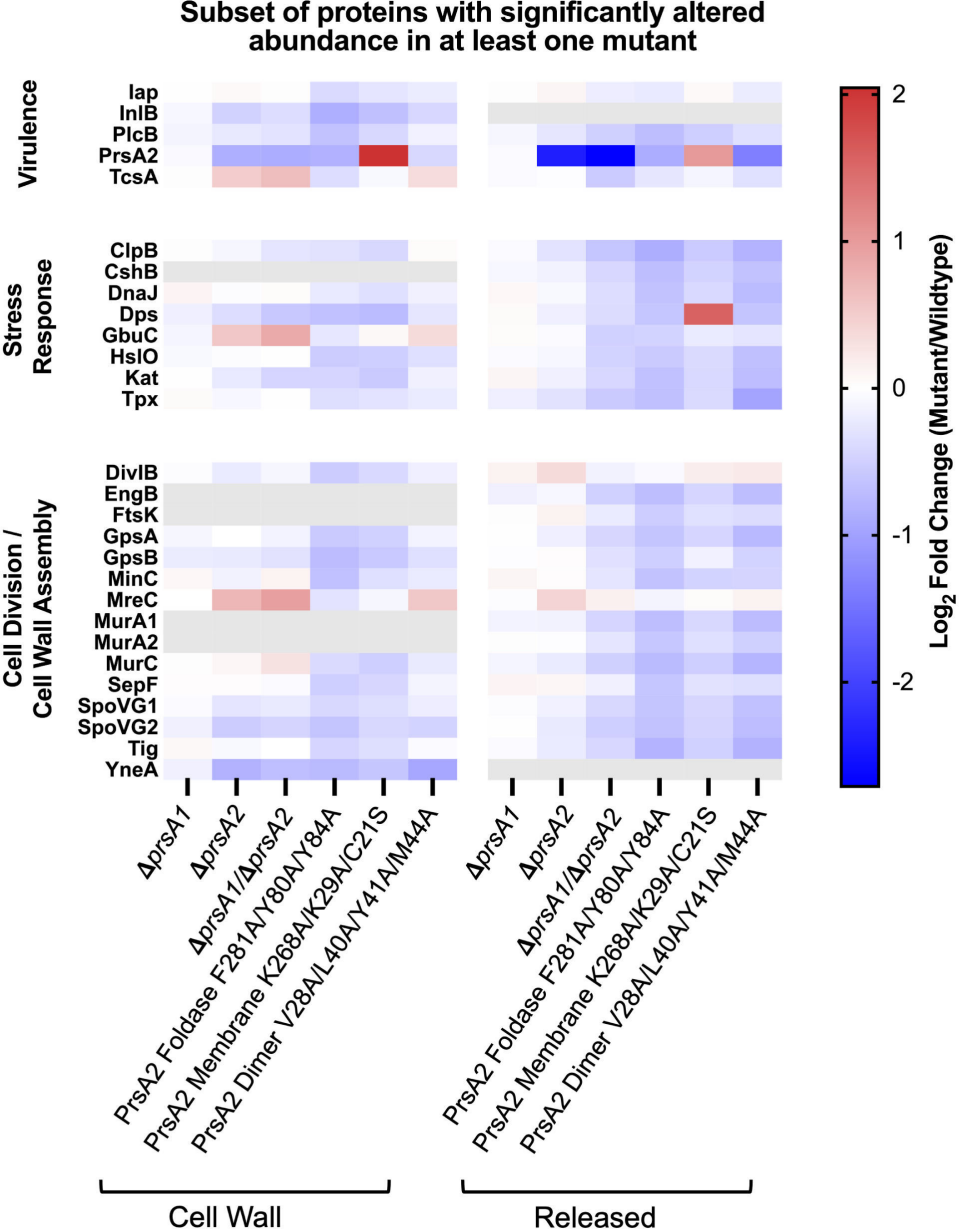
Characterization of *L. monocytogenes* secretion chaperone mutant secretome profiles. TMT-MS analyses of a subset of proteins with significantly altered secretion abundance in at least one prsA mutant are represented as a heat map. (All proteins with significantly altered secretion abundance in at least one mutant are shown in Fig. S1). The relative amounts of the *L. monocytogenes* mutant strain cell wall and released protein fractions with significantly altered secretion levels in at least one strain, as compared to wild type, are shown. A range of fold changes (Log_2_ of mutant/wild type) is represented where a negative fold change is dark blue, a positive fold change is dark red, and no change is white. Proteins not detected in a fraction are gray. Protein fold changes are depicted as an average of two independent experiments.

**Fig 2:**
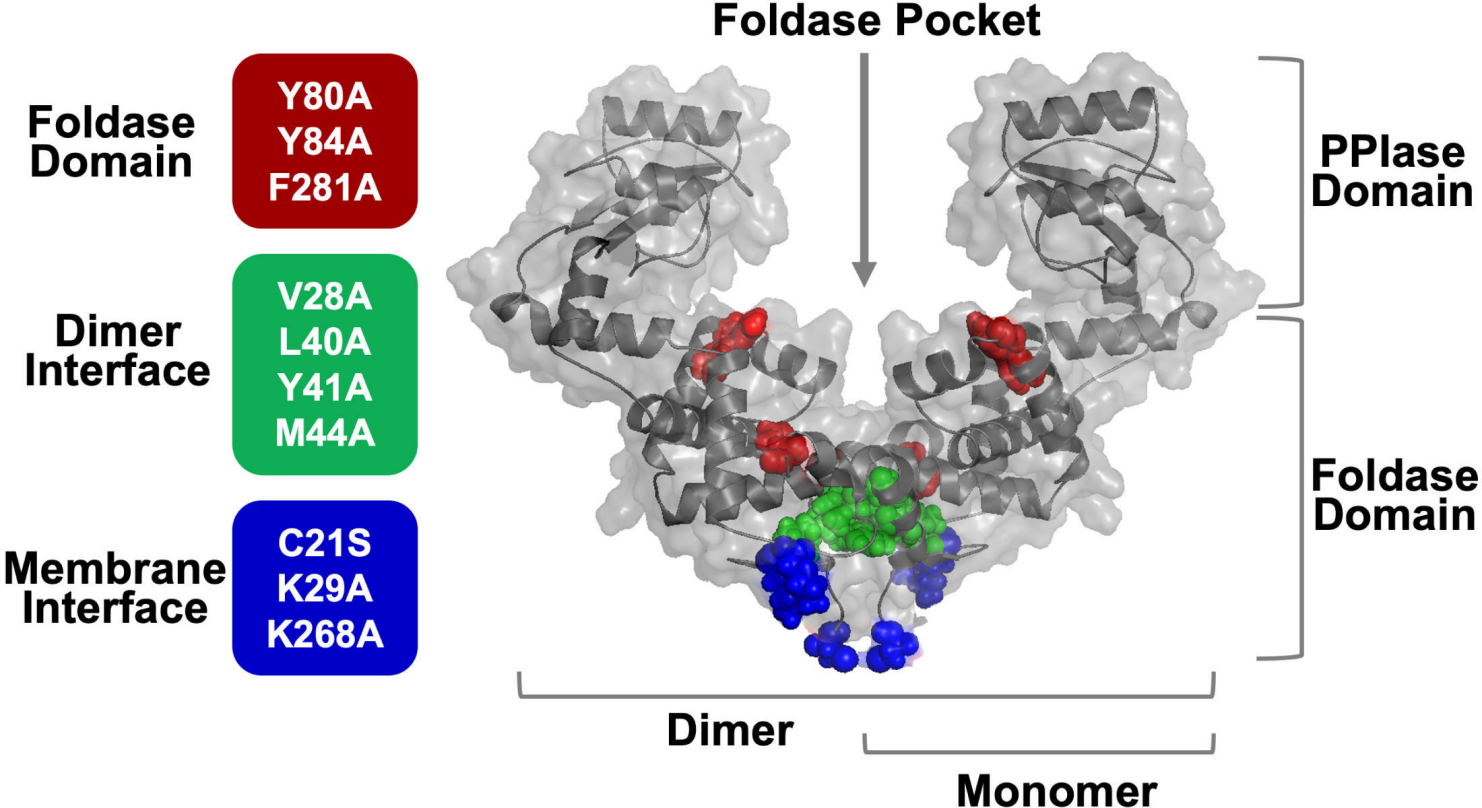
Structural representation of PrsA2 domain and interface variants tested. Transparent surface representation of PrsA2 structure modeled using AlphaFold (29) (previously shown in (17) with selected variant residues depicted as color-coded spheres. The PrsA2 dimer, monomer, PPIase domain, foldase domain, and foldase pocket are indicated. Residues substituted in PrsA2 are grouped and color-coded by domain or interface feature (left). The location of foldase domain substitutions (brick red), dimer interface substitutions (green), and membrane interface substitutions (blue) is indicated by spheres.

To gain a deeper understanding of the spectrum of proteins that require the secreted chaperones PrsA1 and PrsA2 for maturation, we performed TMT-MS analysis on *L. monocytogenes* D*prsA1*, Δ*prsA2,* and Δ*prsA1/*D*prsA2* mutants, and we assessed the alterations in relative secreted protein abundance in cell wall and released (supernatant) fractions (Fig. S1). To further compare how different functional domains of PrsA2 influence substrate stability, TMT-MS analysis was utilized to assess the secretion profiles of previously characterized *L. monocytogenes* PrsA2 structural variants with amino acid substitutions targeting the foldase domain, membrane interface, and dimer interface (Fig. 1; Fig. S1 and S2) (16). Several aromatic residues within the foldase pocket were selected based on their ability to interrupt the foldase activity of PrsA2 by disturbing potential pi-stacking interactions with substrates (16). Dimerization of PrsA2, which forms the foldase pocket, was inhibited by mutating hydrophobic residues at the dimer interface (Fig. 2) (16). Although *L. monocytogenes* PrsA proteins appear to sometimes be released from the bacteria (13, 26), they typically undergo lipid modification of C21, which tethers them to the bacterial cell membrane (18). This residue, in addition to positively charged residues presumed to interact favorably with the negatively charged head groups of cell membrane phospholipids (27, 28), was targeted to inhibit PrsA2 membrane localization (Fig. 2) (16). To ensure bacterial viability, live-dead assays were performed prior to TMT-MS (Fig. S2A). Then, to compare the TMT-MS independent replicates, we calculated Pearson’s correlation coefficient, which showed a positive correlation between replicates in both cell wall and released fractions (Fig. S2B).

**Fig 3:**
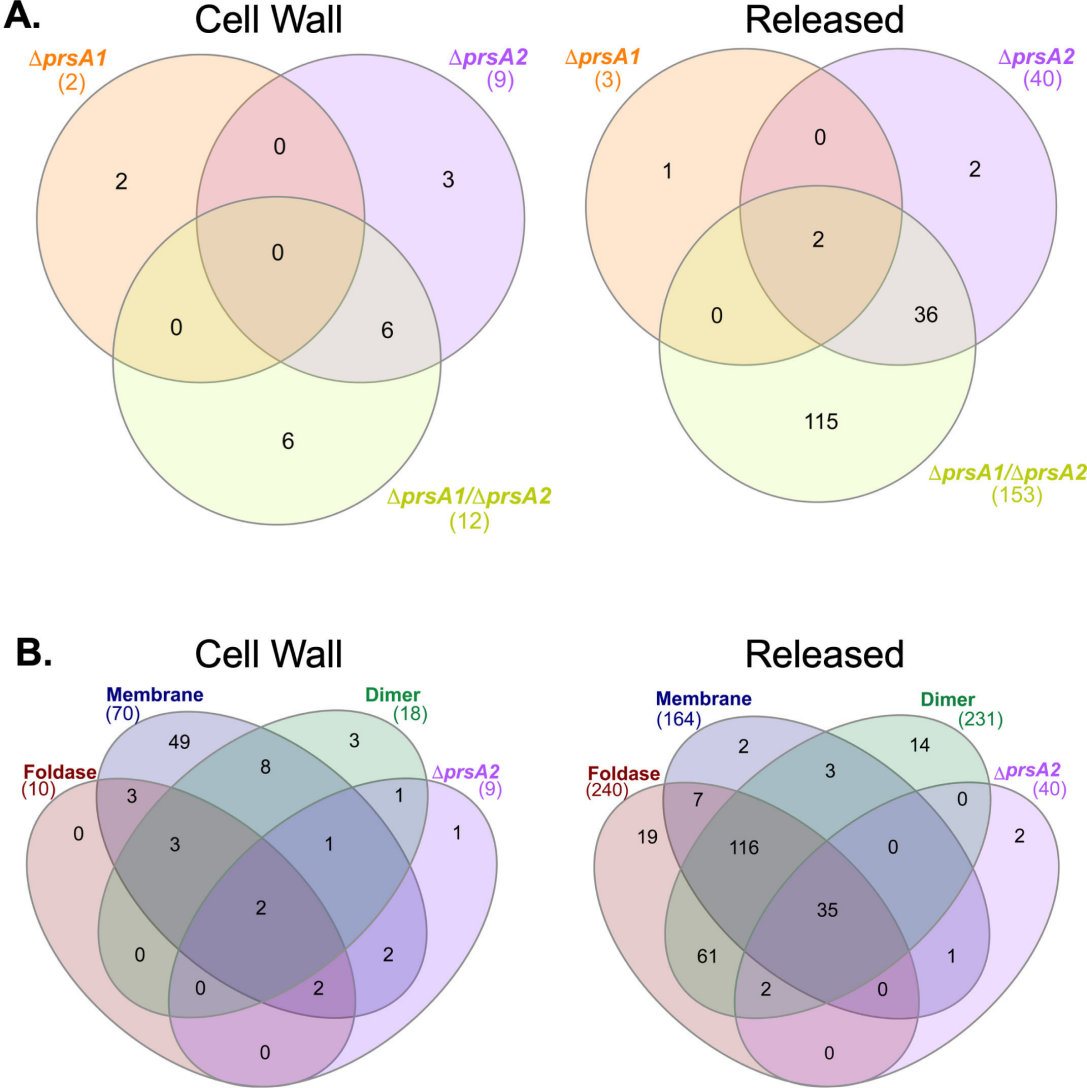
Secretome profiles of proteins significantly altered in *L. monocytogenes* secretion chaperone mutants. Venn diagram representations of proteins, which demonstrated significantly altered secretion levels in cell wall (left) and released fractions (right) of Δ*prsA1* and Δ*prsA2* single and double deletion mutants (**A**) or PrsA2 foldase domain, membrane interface, and dimer interface mutants (**B**). Statistical significance is assessed as *P* ≤ 0.05 by Student’s two-tailed T-test of mutant strains as compared to wild type.

In the cell wall and released fractions, 387 unique proteins were detected in the TMT-MS analyses of *L. monocytogenes* wild type, *prsA1* and *prsA2* single and double deletion mutants, and PrsA2 structural variants (Table S4). Of these, 275 proteins demonstrated statistically significant alterations of their secretion abundance (detectable levels) in one or more PrsA mutant or structural variant (Fig. 1 and 3; Table S1; Table S2). Of the proteins with significantly altered secretion abundance in cell wall fractions of Δ*prsA2* and D*prsA1/*D*prsA2* mutant strains and all PrsA2 structural variants, at least 90% demonstrated a significant decrease when compared to wild type (Fig. S1 and Table S2). Similarly, proteins with significantly decreased secretion abundance accounted for at least 95% of all proteins with significantly altered secretion abundance in the released fractions of Δ*prsA2* and D*prsA1/*D*prsA2* mutant strains and all PrsA2 structural variants (Fig. S1 and Table S2). Venn diagrams depicting the overlap of proteins with significantly altered secretion abundance in each fraction of the Δ*prsA1* and Δ*prsA2* single and double mutants (Fig. 3A) or the Δ*prsA2* mutant and the PrsA2 structural variants (Fig. 3B) suggest both shared and unique contributions of PrsA1 and PrsA2 as well as shared and unique contributions of PrsA2 domains in addition to PrsA2 localization. Interestingly, among the 36 proteins with significantly altered secretion abundance in the released fractions of the Δ*prsA2* and Δ*prsA1/*D*prsA2* mutants and the 35 proteins with significantly altered secretion abundance in the released fractions of Δ*prsA2* and all PrsA2 structural variants, there is an overlap of 34 proteins (Table S3). This group of 34 included proteins such as virulence protein, ClpB (30), cell wall assembly protein, MurA (31), putative cell division proteins, SpoVG1 and SpoVG2 (32), and oxidative stress resistance proteins, Kat and Tpx (33, 34). These data provide insight into the proteins and cellular processes influenced by PrsA1 and PrsA2 chaperone activity and further suggest the importance of the proper PrsA2 foldase domain activity, dimerization, and membrane localization in *L. monocytogenes* protein secretion.

### Gram-positive *prsA* homolog deletion mutants have significantly altered abundance of proteins involved in critical cell processes

**Fig 4:**
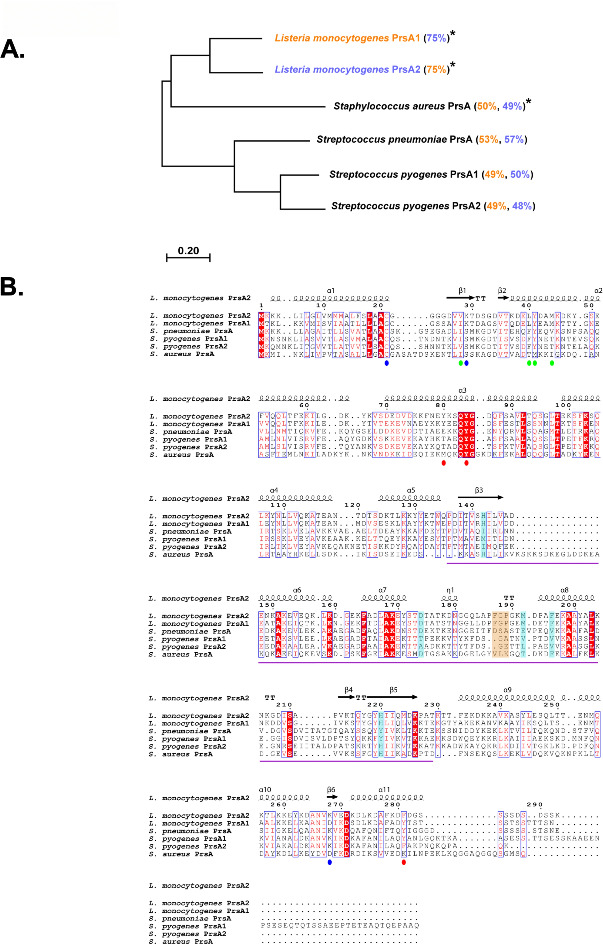
The evolutionary relationship and conservation of PrsA homologs. (**A**) Neighbor-joining phylogenetic tree of PrsA homologs from gram-positive bacteria, created in MEGA using CLUSTAL analysis (46). PrsA homologs that demonstrate PPIase activity are indicated by asterisks. The percent similarity as compared to *L. monocytogenes* PrsA1 (orange) and PrsA2 (purple) is indicated in parentheses. The scale bar indicates one substitution for every 10 amino acid residues. (**B**) A multiple sequence alignment of PrsA homologs from *L. monocytogenes* (PrsA1; WP_003721949.1 and PrsA2; WP_010989927.1), *S. pneumoniae* (PrsA; WP_000727952.1), *S. pyogenes* (PrsA1; WP_002984003.1 and PrsA2; WP_010922719.1), and *S. aureus* (PrsA; WP_000782121.1) was generated in PROMALS3D (47) and the figure was rendered using ESPRIPT (48). The secondary structural elements of *L. monocytogenes* PrsA1 (PDB 5HTF) are displayed above the sequence. The residues substituted in PrsA2 structural variants targeting the foldase domain and membrane and dimer interfaces are marked by red, blue, and green spheres, respectively.

PrsA family proteins are well-conserved with homologs having been identified in diverse gram-positive bacteria (35–43). While much still remains to be learned regarding the full requirements of PrsA for the folding and activity of its predicted substrate proteins, a handful of groups have employed quantitative proteomic methods to investigate changes in the secretome of *prsA* homolog deletion mutants of other gram-positives, including *Streptococcus pneumoniae* D*prsA* (24), *Streptococcus pyogenes* D*prsA1,* D*prsA2, and* D*prsA1*/Δ*prsA2* (25), and *Staphylococcus aureus* D*prsA* (23). A comparison of the amino acid sequences of each of these PrsA homologs reveals that *L. monocytogenes* PrsA2 is most similar to *L. monocytogenes* PrsA1 (75% similarity), followed by *S. pneumoniae* PrsA (57%) and least similar to the *S. aureus* PrsA (49%) and *S. pyogenes* PrsA1 (50%) and PrsA2 (48%) (Fig. 4A). Similarly, after *L. monocytogenes* PrsA2, *L. monocytogenes* PrsA1 is most similar to *S. pnuemoniae* PrsA (53%) but is slightly more similar to *S. aureus* PrsA (50%) than *S. pyogenes* PrsA1 and PrsA2 (both 49%) (Fig. 4A). Interestingly, even though *L. monocytogenes* PrsA1 and PrsA2 have a high similarity to *S. pneumoniae* PrsA, PrsA-associated PPIase activity has only been demonstrated for *L. monocytogenes* PrsA1 and PrsA2, and *S. aureus* PrsA (44, 45).

Aligning the amino acid sequences of these PrsA homologs indicates that there are regions of conservation and variation throughout (Fig. 4B). With respect to the *L. monocytogenes* PrsA2 structural variants, the PrsA2 foldase domain position Y84 is conserved in each of these distantly related PrsA homologs and position F281 is maintained aromatic except in *S. aureus* PrsA (Fig. 4B). Comparison of the *L. monocytogenes* PrsA2 membrane interface variants shows conservation of the lipidated cysteine at position 21 (Fig. 4B). However, the membrane interface positively charged lysines are more variable such that *L. monocytogenes* PrsA homolog position K29 is located at position 31 in other homologs; similarly, position K268 is a lysine for some homologs but position 270 for others (Fig. 4B). Finally, comparison of the *L. monocytogenes* PrsA2 dimer interface demonstrates that position V28 is maintained hydrophobic where some homologs have a valine at this position and others an isoleucine (Fig. 4B). The *L. monocytogenes* dimer interface position Y41 is conserved in all PrsA homologs except *S. aureus,* where only hydrophobicity is maintained as this position is substituted with a methionine (Fig. 4B). With these features in mind, we performed a meta-analysis of the secretomes of each of these PrsA homolog mutants to identify common proteins and protein functions significantly altered by the deletion of the PrsA chaperones.

We observed that several similar pathways were impacted by the loss of PrsA in these distantly related gram-positive bacteria. Interestingly, for *L. monocytogenes,* the impact of PrsA1 is only appreciated when both *prsA1* and *prsA2* are absent. We observed that there were significant disruptions in the secretion of several virulence and immunomodulatory factors across the *prsA* homolog deletion mutants in *L. monocytogenes*, *S. pneumoniae*, *S. pyogenes,* and *S. aureus*. Our *L. monocytogenes* D*prsA2* and Δ*prsA1/*D*prsA2* cell wall fraction TMT-MS analysis found significantly increased secretion abundance of CD4+ T-cell-stimulating antigen TcsA (Fig. 1; Fig. S2). Additionally, phospholipase B (PlcB) has significantly decreased abundance in released fractions of *L. monocytogenes* D*prsA1/*D*prsA2* (Fig. 1; Fig. S2). PlcB contributes to *L. monocytogenes* virulence by facilitating the pore-forming activity of Listeriolysin O (LLO) (49, 50), a cholesterol-dependent, pore-forming toxin, and major *L. monocytogenes* virulence factor required for escape from the phagosomal vacuole (51). Moreover, TMT-MS analysis of a Δ*prsA2* mutant in a hypervirulent *L. monocytogenes* strain revealed that several additional key virulence factors, including LLO, were secreted at significantly altered levels (Table 1, italic text) (17). The secretion of host cell adhesion and entry protein internalin B (InlB) was also significantly decreased in cell wall fractions of *L. monocytogenes* D*prsA2* and Δ*prsA1/*D*prsA2* (Fig. 1; Fig. S2). Another protein of note is enolase (Eno), which is involved in glycolysis but also has a moonlighting role in host cell adhesion (52–56), and has been shown in *S. pneumoniae* to contribute to complement evasion (57). We observed that *S. pneumoniae* Δ*prsA* and *S. pyogenes* D*prsA2* and Δ*prsA1/*D*prsA2* strains similarly exhibited significant reductions in the secretion abundance of Eno (24, 25) (Table 1). In *L. monocytogenes*, Eno was previously found to be absent by two-dimensional (2D) gel analysis of the Δ*prsA2* mutant but present in the wild-type strain (22). *S. pyogenes* virulence factors known to inhibit central host complement proteins and promote infection, including EndoS, PepO, Plr, and ScpA (58–61), demonstrated significantly decreased secretion abundance in the released fractions of one or more of its PrsA homologs (Table 1). *S. pyogenes* SpeB and *S. aureus* SplB, which have both been shown to inhibit immunoglobulin/complement-mediated opsonophagocytosis (59, 62), had significantly increased secretion abundance in the absence of their respective PrsA homolog(s) (Table 1). These data underscore the important contributions of PrsA homologs to the secretion of proteins required for the virulence, pathogenesis, and immunomodulation abilities of several gram-positive pathogens.

**TABLE 1:** Proteins of shared cellular function with significantly altered abundance in PrsA homolog deletion mutants^*a*^

	*L. monocytogenes*		*S. pneumoniae*	*S. pyogenes*			*S. aureus*
	Δ*prsA2*	Δ*prsA1*/Δ*prsA2*	Δ*prsA*	Δ*prsA1*	Δ*prsA2*	Δ*prsA1*/Δ*prsA2*	Δ*prsA*
Virulence/ Immunomodulation	ClpB, TcsA *ActA*, *Iap*, *InlA*, *IsdG*, *LLO*, *Mpl*, *PlcA*, *PlcB*, *Rex*	ClpB, PlcB, TcsA	PsaA	CpsY, HylA, Isp2, Nga, PepO, Pgk, Rgg, RofA, ScpA, SLO, SpeB, SpyCEP	Emm, EndoS, Nga, PepO, Pgk, Rgg, ScpA, SLO, Spd, Spd3, SpeA2, SpeB, SpyCEP	CpsY, Hyl, Irr, Isp, Isp2, LytR, Nga, PepO, Pgk, Plr, Rgg, RofA, ScpA, SibA, SLO, Spd, Spd3, SpeA2, SpeB, SPYcep	Hlb, Hld, HigC, IsdB, SAOUHSC 00258, SAOUHSC 00269, SAOUHSC 01114, SplB, SplF, SspA, SspB
Host cell adhesion/ entry	InlB	InlB	**Eno**, PsaA		**Eno**	**Eno**	Eap, SAOUHSC 00818, SasG, SdrD
Cell division/cell wall assembly	DivIB, GpsA, GpsB, MreC, **MurA2**, SpoVG1, SpoVG2	**EngB**, GpsA, MurA1, **MurA2**, **MurC**, SpoVG1, SpoVG2, Tig	**MltG**, ScpA, **SepF**	DltA, **EngB**, FtsA, FtsE, **MurZ**, **Pbp1A**	DltA, **EngB**, **FtsA**, MapZ, **MurZ**	DacA1, Ddl, DltA, **EngB**, FtsA, FtsE, **MurZ**, **Pbp1A**, **Pbp1B**, **Pbp2A**	EzrA, FmtA, FtsH, **Pbp1**, SAOUHSC 00872, SceD, UppP, XerC
Oxidative stress resistance	Dps, **Kat**, Tpx	Dps, **HslO**, **Kat**, Tpx, TrxB	**HslO**, PsaA, **SodA**		AhpC	AhpC	IsdB, **KatA**, **SodM**
Thermal stress resistance	ClpB	ClpB, **CshB**, GbuC		CshB	GroL	**CshA**, **CshB**, GroL	
Protein secretion		**SecA1**	**SecA2**			**SecA**, **SecY**	Asp1

^*a*^ Proteins with significantly altered secretion abundance identified via TMT-MS analysis of *prsA *homolog deletion mutants in *L. monocytogenes* (this work), *S. pneumoniae* (24) and *S. pyogenes* (25), and ITRAQ LC-MS/MS analysis *S. aureus* (23). Protein homologs presented in bold demonstrated significantly altered abundance in multiple gram-positive *prsA* homolog deletion mutant strains. Proteins listed in italic text demonstrated significantly altered secretion abundance in TMT-MS analysis of *prsA*2 deletion mutants of hypervirulent *L. monocytogenes* strain, prfA* (17).

We found that the deletion of PrsA homologs also significantly disrupted the secretion of proteins involved in cell division and cell wall assembly. There were several proteins involved in these functions that had altered secretion, including EngB, a protein predicted to contribute to cell division and the maintenance of normal cell septation, which demonstrated significantly reduced secretion in *L. monocytogenes* D*prsA1*/D*prsA2* and significantly increased secretion in *S. pyogenes* D*prsA1,* D*prsA2,* and D*prsA1*/Δ*prsA2* (Table 1; Table S2). Crucially, the secretion of penicillin-binding proteins (Pbps), which are required for cell wall assembly and are targets for penicillin and beta-lactam antibiotics (63–68), was significantly increased in released fractions of *S. aureus* D*prsA* and *S. pyogenes* D*prsA1* and D*prsA1*/Δ*prsA2*. In *L. monocytogenes*, three Pbps were identified by 2D gel analysis in the wild-type strain that were absent in the *prsA2* deletion mutant (22). The data provide evidence for the conserved role of PrsA homologs in the secretion of proteins involved in gram-positive bacterial cell division and cell wall assembly, particularly Pbps, highlighting PrsA homologs as potential targets for therapeutic action against gram-positive pathogens.

PrsA homologs were also found to contribute to the secretion of proteins involved in resistance to oxidative stress (Table 1). The abundance of proteins required for resistance to thiol-specific oxidative stress was significantly altered, including superoxide dismutase homologs SodA and SodM, which were increased in released fractions of *S. pneumoniae* D*prsA* and decreased in cell wall fractions of *S. aureus* D*prsA*, respectively (69, 70). Furthermore, in released fractions of *L. monocytogenes* D*prsA2* and Δ*prsA1*/D*prsA2* mutants, the abundance of Tpx, a predicted thiol peroxidase (71), in addition to TrxB, a predicted thioredoxin reductase (72) in Δ*prsA1*/D*prsA2* mutants, was significantly reduced (Table 1; Table S2). In *L. monocytogenes,* D*prsA2* and Δ*prsA1*/D*prsA2* released fractions*,* and *S. aureus* D*prsA* cell wall fractions, the hydrogen peroxide-specific oxidative stress resistance protein catalase homologs, Kat and KatA (71, 73), also showed significantly decreased secretion abundance. Likewise, the chaperonin HslO, which also protects against oxidative stress (74), was found to have significantly decreased secretion abundance in *L. monocytogenes* D*prsA1/*D*prsA2* released fractions and *S. pneumoniae* D*prsA* cell wall fractions ( Table 1; Table S2). These findings, in addition to the evidence that the absence of PrsA homologs causes significant alterations in the secretion of proteins required for thermal stress resistance in *L. monocytogenes* and *S. pyogenes*, and acidic stress resistance in *S. pyogenes* (Table S2), suggest that gram-positive PrsA homologs have critical roles in bacterial resistance to a variety of environmental stressors.

Finally, we also note that the secretion of critical components (75, 76) of the Sec secretion pathway was significantly increased in released fractions of *S. pneumoniae* D*prsA* and *S. pyogenes* D*prsA1*/Δ*prsA2* (Table 1) while SecA1 was significantly decreased in released fractions of *L. monocytogenes* D*prsA1*/D*prsA2* (Table 1; Table S2). In cell wall fractions of *S. aureus* D*prsA* mutants, Asp1, which acts as an important accessory component to its Sec secretion pathway (77), demonstrated significantly decreased secretion abundance (Table 1). Although these bacteria vary in genome and predicted proteome sizes (Table S3), they each rely on the Sec pathway for the secretion of a variety of proteins (75, 76). Bioinformatic approaches suggest that *L. monocytogenes* and *S. aureus* strains secrete approximately 25% and 28% of their proteomes, respectively (78, 79), while *S. pneumoniae* and *S. pyogenes* strains secrete approximately 12% and 13% of their proteome, respectively (80, 81). Taken together, these data support the hypothesis of the conservation of PrsA homologs across gram-positive bacteria being influenced by the crucial role these chaperones play in the secretion of several key proteins involved in critical functions from bacterial physiology to virulence. These PrsA homologs retain the ability to fold client proteins, including homologous client proteins, despite variations in their amino acid sequence (Fig. 4B), suggesting that their function as chaperones may primarily rely on the retention of a few key residues.

### PrsA2 contributes to *L. monocytogenes* oxidative stress resistance

We found that secretion of oxidative stress resistance proteins Dps, HslO, Kat, Tpx, and TrxB (33, 34, 72, 74, 82) was significantly decreased in *L. monocytogenes* D*prsA2* and/or Δ*prsA1*/D*prsA2* mutants and in all three PrsA2 structural variants (Table 1; Table S1; Table S2). Likewise, homologs of some of these oxidative stress resistance proteins also showed significantly decreased secretion levels in *S. pneumoniae* and *S. aureus* D*prsA* mutants. This led us to hypothesize that PrsA homologs contribute to bacterial resistance to oxidative stress. To test our hypothesis, *L. monocytogenes* strains were challenged with a variety of oxidative stress-inducing agents, including copper chloride and cadmium chloride, which induce redox-active stress, hydrogen peroxide, which acts as a direct oxidant, and diamide, which induces thiol-specific oxidizing damage.

**Fig 5:**
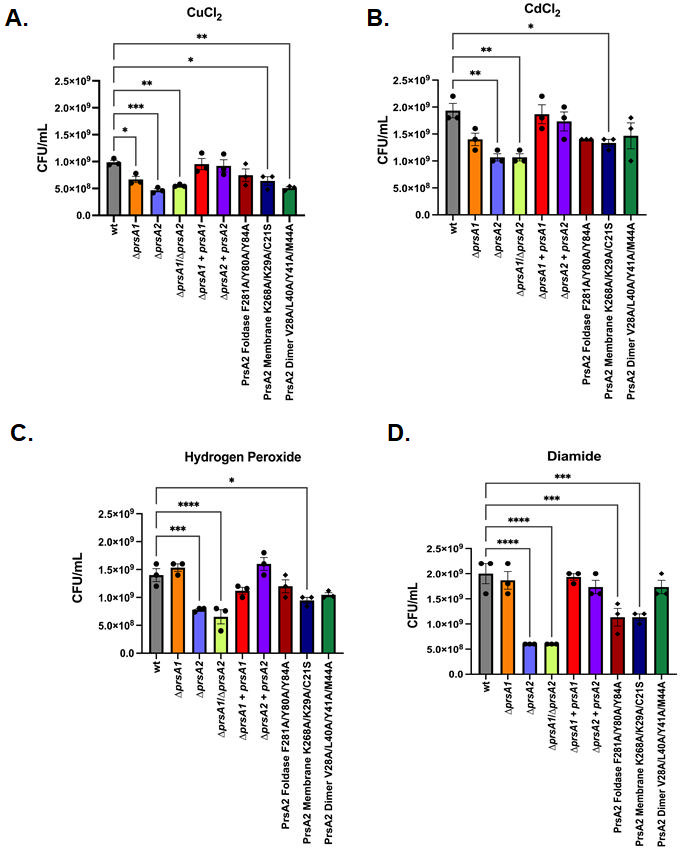
The contribution of *L. monocytogenes* secretion chaperones to oxidative stress resistance. Bacterial growth is shown as colony-forming units (CFU) per mL of strains grown to early log phase then serial diluted and spot plated onto brain heart infusion (BHI) agar with 0.75 mM copper (II) chloride (**A**), 0.025 mM cadmium chloride (**B**), 4 mM hydrogen peroxide (**C**), or 1.25 mM diamide (**D**) and grown overnight at 37°C. Error bars represent the standard error of the mean for three independent experiments. Asterisks indicate statistical significance of *P* ≤ 0.05 by ordinary one-way ANOVA and Dunnett’s multiple comparisons test, with a single pooled variance.

The Δ*prsA2* and Δ*prsA1*/D*prsA2* mutants were significantly less resistant to oxidative stress regardless of oxidizing agent when compared to the wild-type strain (Fig. 5). Redox-active stress induced by copper chloride also inhibited the growth of the Δ*prsA1* mutant as compared to wild type (Fig. 5A). The susceptibility of Δ*prsA1* and D*prsA2* strains to oxidative stress was fully complemented by the introduction of *prsA1* and *prsA2*, respectively, in single copy on the integrated plasmid vector pPL2 (Fig. 5). Although the membrane localization of PrsA2 appears to be required for resistance to oxidative stress caused by all tested oxidizing agents (Fig. 5), the PrsA2 dimer and foldase variants only showed increased susceptibility to copper chloride-induced redox-active stress and diamide-induced thiol-specific oxidative stress, respectively (Fig. 5A and D). Taken together, while PrsA2 is required for *L. monocytogenes* resistance to oxidative stress, the contributions of its functional domains depend on the oxidizing agent and/or the type of oxidative stress the bacteria encounter. Notably, PrsA1 is more specific than PrsA2 and was only observed to contribute to redox-active stress induction by copper chloride.

### PrsA2 contributes to *L. monocytogenes* osmotic stress resistance and is required for cold-osmotic stress resistance

The absence or mutation of PrsA2 also significantly affected the secretion levels of proteins implicated in resistance to thermal stress, including glycine betaine/carnitine transport protein, GbuC (83, 84), which was significantly increased in cell wall fractions of Δ*prsA1/*D*prsA2* mutants and PrsA2 dimer structural variant compared to wild type (Fig. 1; Table S2). GbuC is encoded by the *gbu* operon and forms part of the GbuABC transporter complex, which, along with BetL and OpuC, is involved in the transport of glycine betaine and carnitine, important osmolytes, into the cell (83–85). These osmolytes protect *L. monocytogenes* protein structure under cold and osmotic stress without interrupting the cellular machinery (83, 85–88). We therefore hypothesized that PrsA2 and PrsA2 dimerization are critical for *L. monocytogenes* resistance to thermal and thermo-osmotic stress. To test this, strains were grown at 42°C, 37°C, 30°C, and 16°C to target a range of temperatures at which the bacteria grow. We chose the heat shock temperature of 42°C, the optimal growth (and host) temperature of 37°C at which *L. monocytogenes* demonstrates optimum growth, the temperature of 30°C at which *L. monocytogenes* is motile and flagellated, in addition to the upper range of home refrigeration temperatures of ready-to-eat meals which is 16°C as determined by a national survey in the United States (2–4).

We found that at 42°C, the Δ*prsA1*, Δ*prsA2,* and Δ*prsA1/*D*prsA2* mutants, and their complements generally all grew similarly to wild type (Fig. S4A). While the PrsA2 structural variants had slower growth after 3 h, the foldase and membrane variants recovered as time went on, leaving only the dimer variant with a lower concentration of cells throughout the exponential phase and eventually during the decline (Fig. S4B). At 30°C and 37°C, all mutants, variants, and complemented strains demonstrated a similar growth rate to wild type (Fig. S4C through F). Likewise, when grown at 37°C in chemically defined media (CDM), the growth rates of the Δ*prsA1* and Δ*prsA2* single and double mutants and PrsA2 structural variants were overall similar to the wild-type strain (Fig. S3). A noticeable gap in growth developed between the wild-type strain and the Δ*prsA2* and Δ*prsA1*/D*prsA2* mutants at 16°C (Fig. S4G), whereas the PrsA2 foldase variant continued to display a similar growth rate to wild type as did membrane and dimer variants (Fig. S4H). These results demonstrate that, at lower temperatures, PrsA2 contributes to the resistance to thermal stress, but foldase activity and membrane localization are not necessary for this function, while the dimerization of PrsA2 contributes to the resistance to heat stress, specifically.

**Fig 6:**
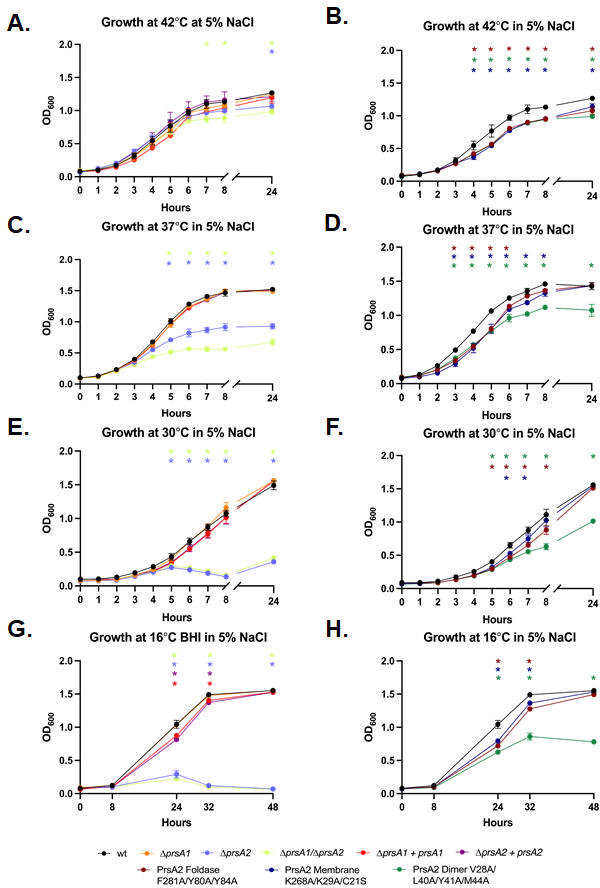
*L. monocytogenes* PrsA2 is critical for osmotic and thermo-osmotic stress resistance. Bacterial growth of the wild type, *prsA2* and *prsA1* deletion mutants and complements (left) and foldase domain, dimer interface, and membrane interface mutants (right) under osmotic stress induced by the addition of 5% NaCl to BHI media. Optical density (OD) at 600 nm was monitored over a period of 24 h at 42°C (**A & B**), 37°C (**C & D**), and 30°C (**E & F**) or 48 h at 16°C (**G & H**). Error bars represent the standard error of the mean for three independent experiments. Asterisks indicate statistical significance of *P* ≤ 0.05 by repeated measures two-way ANOVA tests and Dunnett’s multiple comparison tests, with a single pooled variance.

We next tested *L. monocytogenes* strains for their ability to survive under thermo-osmotic stress. The Δ*prsA1/*D*prsA2* mutant demonstrated significantly reduced growth starting at 6 h under thermo-osmotic stress at 42°C while the Δ*prsA2* mutant only showed decreased cell concentration at the 24-h mark, and the Δ*prsA1* mutant and PrsA1 and PrsA2 complements grew similarly to wild type (Fig. 6A). The PrsA2 structural variants were also more susceptible to thermo-osmotic stress at 42°C showing significant growth decreases starting after 4 h of growth and continuing throughout the time course (Fig. 6B). Under osmotic stress at 37°C, the Δ*prsA2* and Δ*prsA1/*D*prsA2* mutants had significantly decreased growth, only getting to about half the maximum optical density (OD_600_) as the wild-type, Δ*prsA1* mutant, and complemented strains (Fig. 6C). The PrsA2 structural variants showed significantly increased susceptibility to osmotic stress at 37°C starting after 3 h of growth, and while the foldase variant showed recovery after 6 h, the membrane variant did not reach wild-type levels until the 24-h time point, and the dimer variant remained reduced throughout (Fig. 6D). The observed growth disparity between wild type and the Δ*prsA2* and Δ*prsA1/*D*prsA2* mutants and the PrsA2 dimer variant continues and widens when the strains are grown under osmotic stress at 30°C (Fig. 6E and F). Under these conditions, the PrsA2 foldase and membrane variants again showed a statistically significant decrease in growth during early to mid-exponential phase, eventually recovering at later time points (Fig. 6F). Growth at 16°C under cold-osmotic stress resulted in the biggest decrease in growth for the Δ*prsA2* and Δ*prsA1/*D*prsA2* mutants, which showed no growth after 32 h (Fig. 6G). The *prsA1* and *prsA2* complemented strains also demonstrated decreased growth at the 24-h and 32-h time point (Fig. 6A), and while constitutive expression of *prsA2* returned growth to wild-type levels, constitutive expression of *prsA1* did not (Fig. S5), suggesting that there may be regulatory factors upstream of *prsA1* required for its role in cold-osmotic stress resistance. Under osmotic stress at 16°C, the PrsA2 foldase and membrane variants showed reduced growth at 24 and 32 h, while the PrsA2 dimer variant had reduced growth throughout, only growing to about half the maximum optical density of wild type (Fig. 6H). These findings illustrate the requirement of PrsA2 for *L. monocytogenes* thermo-osmotic stress resistance and associate PrsA2 foldase activity and membrane localization together with a role for dimerization as important in this context.

### The contributions of *L. monocytogenes* PrsA homologs to cell division

**Fig 7:**
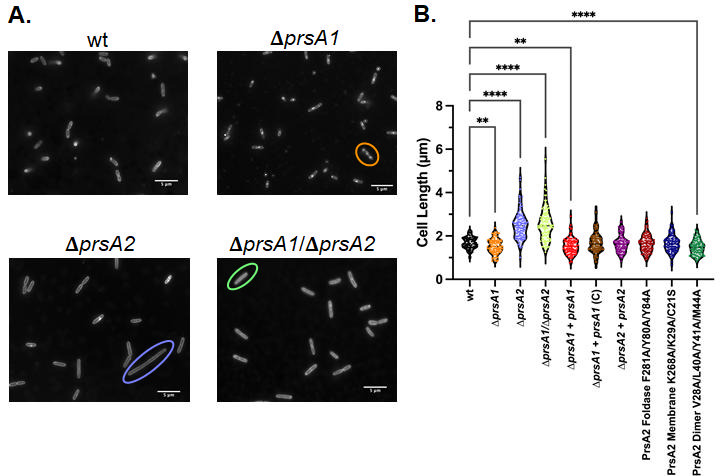
The contribution of *L. monocytogenes prsA2* to cell length. (**A**) Representative microscopy images of Nile red-stained wild type, Δ*prsA1*, Δ*prsA2,* and Δ*prsA1*/Δ*prsA2* mutants (OD_600_ ~ 1.2) are shown, where examples of elongated cells are circled. (**B**) Violin plots depicting the quantification of phase contrast microscopy analysis of the average length of *L. monocytogenes* cells. Phase contrast microscopy analysis was performed in duplicate for all samples, *N* = 100 cells for all samples. Cell length was quantified using ImageJ (92). Asterisks indicate statistical significance of *P* ≤ 0.05 by Brown-Forsythe and Welch ANOVA tests, and Dunnett’s T3 multiple comparisons test, with individual variances computed for each comparison. Error bars represent the standard error of the mean. Images were taken with a 100× oil objective, scale bars represent 5 µm as labeled.

Several cell division and cell wall assembly proteins demonstrated altered secretion in the absence of PrsA2, including DivIB and MreC, which had significantly increased secretion abundance in released fractions of Δ*prsA2* mutant strains (Fig. 1; Table S2). DivIB has been shown to stabilize or promote the assembly of the division complex in other gram-positive bacteria, while MreC has been shown to contribute to cell shape, with critical elongasome function in rod-shaped bacteria (89–91). We therefore investigated whether the observed change in secretion abundance of cell division and cell wall assembly proteins affected the morphology of *L. monocytogenes* (Fig. 7; Fig. S6).

During the early stationary phase (OD_600_ ~1.2), the lengths of cells in the Δ*prsA2*, Δ*prsA1/*D*prsA2* strains were found to be significantly longer than wild-type cells by an average of 1.5× (Fig. 7; Fig. S6B). This phenotype was lost in the *prsA2* wild-type complemented strain, which had similar average cell lengths as the wild-type strain (Fig. 7B; Fig. S6B). Targeted cell membrane staining revealed that while there was no significant difference in the occurrence of septa formation between wild type, Δ*prsA1*, Δ*prsA2*, Δ*prsA1/*D*prsA2*, or the complements (Fig. S6A), the deletion of Δ*prsA1* and/or Δ*prsA2* led to aberrations to cell division resulting in elongated cells (Fig. 7A, Δ*prsA2* circle) or the formation of multiple septa but no cell separation (Fig. 7A, Δ*prsA1* circle). The PrsA2 dimer variant exhibited cells with slightly shorter average lengths than wild type, while the PrsA2 foldase and membrane variant strains had a similar average cell length (Fig. 7B). While complementing *prsA1* with its native promoter did not relieve the phenotype of shorter cell length observed in the Δ*prsA1* mutant strains, we found that *prsA1* complementation with a constitutively active promoter resulted in cell lengths similar to the wild-type strain (Fig. 7B). Interestingly, when cells were analyzed during mid-exponential growth phase (OD_600_ ~ 0.6), the enhanced cell length of Δ*prsA2* strains, while still present, is more subtle, and no defects in cell morphology were observed for the other *prsA* mutants or complemented strains (Fig. S7). This subtle increase in cell length of the Δ*prsA2* mutant at mid-exponential growth phase (Fig. S7) and the observed reduction in cell length of the Δ*prsA1* strains during early stationary phase (Fig. 7; Fig. S6) suggests that the opposing phenotypes of these mutations have a neutralizing effect in the Δ*prsA1/*D*prsA2* strains during the mid-exponential growth phase, resulting in an average cell length similar to that of wild type (Fig. S7B). A comparison of the average cell length of most strains at each imaged time point revealed a trend of longer average cell lengths during mid-exponential phase versus early stationary phase, except in the Δ*prsA2* and Δ*prsA1/*D*prsA2* strains, where the opposite was observed (Fig. S8A).

Additionally, in early stationary phase images of Δ*prsA1* strains, cells were observed to be wider than wild-type strains (Fig. 7B). Indeed, an assessment of total cell area revealed that while the Δ*prsA1* strains were, on average, shorter than wild type, the average cell area of the two strains was as similar (Fig. S8B). The average cell area for the remaining mutant strains and complements mirrored the results of the cell length analysis at both tested growth phases (Fig. S8B and C). A trend of larger area during the mid-exponential phase, when compared to early stationary phase, was also observed in most tested strains, except Δ*prsA1/*D*prsA2* strains, which had larger average areas during early stationary phase, and Δ*prsA2* strains, which showed no significant difference in area between growth phases (Fig. S8D). These data establish a novel role for PrsA1 in the maintenance of *L. monocytogenes* cell morphology and expand on the previously observed critical role of PrsA2 in *L. monocytogenes* cell wall integrity by providing evidence of an additional role in cell division. While the dimerization of PrsA2 makes a small contribution to this role, it does not appear to be dependent on PrsA2 foldase activity or localization to the cell membrane.

## DISCUSSION

PrsA1 and PrsA2 are secretion chaperones with critical roles in protein folding in the *L. monocytogenes* membrane-cell wall compartment. Here, we utilized TMT-MS for an extensive proteomic analysis of the contributions of PrsA1 and PrsA2 to the *L. monocytogenes* secretome and, based on our findings, performed phenotypic assays and microscopy to identify novel roles for both chaperones in *L. monocytogenes* stress resistance and cell length and shape maintenance. PrsA2 was first characterized in *L. monocytogenes* as a key contributor to intracellular growth and cell-to-cell spread (93). It was then observed to have increased secretion in strains with the constitutively expressed transcriptional activator *prfA* (94). Later, PrsA2 was determined to be a critical secretion chaperone required for virulence (19, 95) with a ring of aromatic residues lining the foldase pocket, making an important contribution to this role (16). PrsA1 and PrsA2 share significant amounts of amino acid homology (58% identity and 75% similarity), and despite differences in their functional capacities, when expressed under the *prsA2* promoter, PrsA1 fully complemented deficiencies in cell wall integrity and osmotic stress resistance observed in Δ*prsA2* mutants to wild-type levels (13). More recently, PrsA1 and PrsA2 were found to function together under the control of the two-component signal transduction system, PieRS, to bolster *L. monocytogenes* survival during intra-gastric infection and translocation across the small intestines to distal organs such as the liver and spleen in mice (20). Our TMT-MS analysis builds on this data and previous hypotheses of *L. monocytogenes* PrsA1 and PrsA2 having a shared client protein repertoire as a large number of proteins were found to have significantly altered secretion abundance in the Δ*prsA1/*D*prsA2* double mutant (Fig. 1 and 3A). Structural comparisons of PrsA1 and PrsA2 have also highlighted differences in the electrostatic nature of their respective PPIase active sites and key amino acid residues in their foldase pockets (16). These biochemical differences suggested that PrsA1 and PrsA2 have a subset of proteins that are uniquely chaperoned by each, a hypothesis further strengthened by our observation of several proteins, which only demonstrated significantly altered secretion abundance in either the Δ*prsA1* strain or the D*prsA2* strain (Fig. 3A; Table S2).

Previous investigations found that while the foldase activity, membrane localization, and dimerization of PrsA2 contribute to its role in *L. monocytogenes* virulence and secretion of the virulence factor, LLO, it is the dimerization of the protein that is most important (16). Dimerization of PrsA2 has also been previously implicated for significant contributions to the chaperone’s role in cell-to-cell spread and resistance to the cell wall-active antibiotic penicillin, in addition to environmental stresses, including osmotic and acidic stress (16). Similarly, PrsA2 foldase activity was shown to contribute to penicillin resistance and play a minor role in cell-to-cell spread (16). Our TMT-MS analysis revealed that several proteins involved in critical processes such as cell division, cell wall assembly, and resistance to oxidative stress had significantly altered secretion abundance in either the absence of *prsA2* or in strains with mutations targeting the foldase activity, membrane localization, or dimerization of PrsA2 (Fig. 1; Fig. S2). The importance of these structural features was highlighted by overall larger decreases in secretion abundance in PrsA2 foldase and membrane structural variants in cell wall fractions and foldase and dimer variants in released fractions compared to Δ*prsA1,* D*prsA2,* and Δ*prsA1/*D*prsA2* mutants (Fig. 1; Fig. S1). This suggests that the absence of PrsA1 and/or PrsA2 may initiate a stress response which mediates some compensatory measures for protein folding, which may include HtrA, the final secretion chaperone working in the cell membrane-cell wall interface of *L. monocytogenes,* which is also well-conserved in mammals, and gram-positive and gram-negative bacteria (96). Previously, Δ*prsA2/*D*htrA* mutants demonstrated larger decreases in bacterial intracellular growth and survival in mouse models of infection than those observed in Δ*prsA2* mutants alone (22). HtrA homologs have also been shown to reduce the accumulation of misfolded proteins that occurs in the absence of PrsA in *B. subtilis* and SurA, an *Escherichia coli* periplasmic PPIase chaperone (97, 98). These fail-safe measures might not be initiated in mutants expressing PrsA2 structural variants, as the chaperone would be present and available for substrate binding, but inefficient folding and activation, resulting in an overall decrease in protein secretion. Together, this analysis corroborates previous findings of PrsA2 dimerization, foldase domain activity, and/or membrane localization, making critical contributions to PrsA2 chaperone function.

Interestingly, our investigations into secretion chaperone roles in oxidative stress resistance revealed a novel role for PrsA1 in resistance to redox-active stress (Fig. 5A). PrsA2 also contributed to resistance to redox-active stress in addition to diamide-induced thiol-specific oxidative stress and direct oxidation by hydrogen peroxide, with its membrane localization playing a significant part in this function (Fig. 5). This newly discovered functional capability of PrsA1 may be linked to its role in translocation across the small intestine as that environment is prone to oxidative stress during inflammation which exerts antimicrobial action that targets bacteria like *L. monocytogenes* (20, 99, 100). Our findings also show that the importance of PrsA2 in *L. monocytogenes* resistance to environmental stress extends to include cold, osmotic, and thermo-osmotic stress and that PrsA2 dimerization often makes a significant contribution to this role (Fig. S4G and S6), further exemplifying that PrsA2 functional domains likely contribute to PrsA2 chaperone activity for different subsets of protein substrates.

Cell division and cell wall assembly are essential processes in the bacterial life cycle. *L. monocytogenes* PrsA2 is known to make significant contributions to cell wall integrity, including resistance to cell wall-active antimicrobials (13). Mutants lacking *prsA1* are more sensitive to cell-wall active antimicrobials, but this effect is only seen when *prsA2* is also absent (16). We found that both PrsA1 and PrsA2 contribute to proper cell morphology in *L. monocytogenes* during the early stationary growth phase (Fig. 7; Fig. S8B). It is tempting to speculate that this phenotype is the result of cell division proteins with significant secretion abundance differences in the Δ*prsA2* and Δ*prsA1*/D*prsA2* mutants, or PrsA2 structural variants, namely, DivIB, FtsK, MreC, and YneA (Fig. 1), especially as mutations involving DivIB and MreC have been shown to disrupt cell division and cause elongation in rod-shaped bacteria (101, 102). Mutations in PrsA homologs have similarly exhibited significant changes in the secretion of cell division and cell wall assembly proteins, and defects in morphology have been observed in *S. pneumoniae, S. pyogenes,* and *Streptococcus suis prsA* homolog deletion mutants and in *B. subtilis* PrsA-depleted cells (24, 25, 42, 103). Altogether, this evidence expands on what is known about the contribution of PrsA to *L. monocytogenes* cell division and cell wall assembly, both in rod-shaped bacteria as well as cocci.

This work is limited by the growth phase at which the bacterial samples were collected for TMT-MS analysis. While our secretome analysis was performed on bacterial samples grown to mid-exponential phase (OD_600_ ~ 0.6), previous work investigating the contribution of PrsA1 and PrsA2 to the *L. monocytogenes* secretome used bacterial samples taken at early stationary phase (OD_600_ ~ 1.2) and identified several proteins not captured in this work such as Pbps which play a critical role in cell wall assembly and susceptibility to cell wall active antimicrobials (22, 44). Recently, we published work where we performed TMT-MS secretome analysis of hypervirulent *L. monocytogenes* strains, prfA*, and of Δ*prsA2* in a prfA* background (OD_600_ ~ 0.6) (17), which were generated to mimic the host infectivity state by constitutively expressing PrfA*,* a transcriptional activator that regulates a virulence regulon containing several genes required for successful host infection including *prsA2* (94, 104, 105). Targeting various *L. monocytogenes* growth phases and physiological states, that is, saprophyte vs. infectious, adds to a more complete understanding of the roles of the PrsA secretion chaperones throughout the lifecycle of the bacterium. Additionally, as biophysical and biochemical investigations have been used to better understand the interaction of PrsA2 with its client protein LLO (17), techniques such as isothermal titration calorimetry and chaperone-assisted folding assays can also be utilized to explore the ability of PrsA1 and PrsA2 to bind, fold, and activate proteins that demonstrated altered secretion levels in their absence to determine whether these proteins are clients of the chaperones. Identifying proteins that directly interact with PrsA1 and PrsA2 will provide insight into the exact perturbations occurring in processes such as stress resistance and cell wall assembly in the absence of the chaperones, thereby expanding our knowledge of PrsA1 and PrsA2 chaperone function and *L. monocytogenes* physiology, with potential implications for PrsA homologs in other gram-positive pathogens.

Our meta-analysis of quantitative proteomic secretome data highlighted the contributions of PrsA homologs to the secretion of proteins involved in critical bacterial processes ranging from virulence, including host cell entry and/or adhesion and immunomodulation, to physiology such as cell division and resistance to several stressors. Furthermore, among the proteins demonstrating significantly altered secretion abundance in the absence of PrsA homologs, there were several potential client protein homologs previously implicated in critical processes, including protein secretion, cell division and cell wall assembly, and resistance to oxidative and thermal stress. This discovery speaks to findings in previous work, which showed that PrsA homologs from several Gram-positive bacteria could complement for a select number of phenotypes associated with the loss of *prsA2* in *L. monocytogenes*. Specifically, *S. aureus* PrsA, *S. pneumoniae* PrsA, and *S. pyogenes* PrsA1 and PrsA2 all completely restored *L. monocytogenes* swimming motility and growth under extremely acidic or basic conditions, and all at least partially complemented osmotic resistance and cell-to-cell spread in Δ*prsA2* strains (13). Together with our findings of the contributions of membrane localization and dimerization to PrsA2 function in resistance to oxidative and thermo-osmotic stress, respectively, our meta-analysis adds to our understanding of the motivating force behind the vast conservation of PrsA homologs in pathogenic and non-pathogenic gram-positives regardless of whether PPIase activity is retained or not. As more research is conducted into PrsA homologs and their client proteins, gram-positive protein secretion, and perhaps its evolution, will be better defined.

The findings of this paper support and expand on previous observations of the varied and important roles PrsA chaperones play in *L. monocytogenes*, particularly in maintaining bacterial physiology and promoting resistance to environmental stressors. Identifying proteins in the *L. monocytogenes* secretome with altered secretion in the absence of PrsA1 and PrsA2 brings us one step closer to defining the client protein repertoires of each chaperone. Because the potential client proteins identified vary both in structure and in function, the secretome analysis of PrsA2 structural variants provides us with PrsA2 structural features that may be required to chaperone different groups of client proteins. The conservation of PrsA homologs across gram-positive bacterial species, in addition to conserved functions shared by these homologs, highlights these chaperones as a potential target for therapeutic interventions against gram-positive pathogens.

## MATERIALS AND METHODS

### Bacterial strains and media

Bacterial strains used in this study are listed in Table S4. *L. monocytogenes* 10,403S is the wild-type (wt) strain. *L. monocytogenes* 10,403S containing an erythromycin resistance gene (*erm*) in place of the *prsA2* coding sequences is referred to as Δ*prsA2* (19); *L. monocytogenes* 10,403S with a *prsA1* in-frame deletion is referred to as Δ*prsA1* (19); and Δ*prsA1* transduced with Δ*prsA2::erm* is referred to as Δ*prsA1*/Δ*prsA2* (22). The Δ*prsA2* mutant was used for complementation with the designated *prsA2* mutant allele. *Escherichia coli* One Shot TOP10 (Invitrogen) and SM10 were used as host strains for recombinant plasmids (16). The integration plasmid pPL2 (106) was used for genetic complementation into *L. monocytogenes* (16). LB and BHI medium were used for the growth of *E. coli* and *L. monocytogenes*, respectively.

### Construction of the *prsA1*-complemented strain

To generate the *prsA1*-complemented strain, we used the previously generated plasmid NF-p3860 (20) (Table S4). Briefly, the region encompassing 195 bp upstream and 66 bp downstream of *prsA1* was amplified from *L. monocytogenes* 10,403S genomic DNA with primers PrsA1UPSacF1 (5′AGTAGAGCTCGACGGGCACAAATTCGAAGT) and PrsA1XmaR2 (5′AGTACCCGGGGCGTGTGTTTTTGACGGGAA) and cloned into the integration plasmid pPL2 (106). The pPL2 plasmid allows for integration at a single neutral site within the *L. monocytogenes* chromosome, and integrated genes are expressed by their native promoter (106). The NF-p3860 plasmid was transformed into *E. coli* SM10 cells and subsequently introduced into the *L. monocytogenes* Δ*prsA1* strain by conjugation. Constructs and strains were verified by DNA sequencing analysis.

### Assessment of bacterial viability

A live-dead bacterial viability kit (Molecular Probes L13152) was used according to the manufacturer’s specifications with minor adjustments. Briefly, bacterial cultures were grown, in duplicate, to OD_600_ ~0.6, washed, and normalized to 4 × 10^7^ bacteria per mL in phosphate-buffered saline (PBS) at pH 7 in duplicate. One set of normalized cells was heat-killed by boiling for 10 min and then chilled on ice. As per the manufacturer’s instructions, five different proportions of live to dead cells were mixed in borosilicate glass tubes and aliquoted into a black 96-well flat-bottom plate, followed by the addition of a 2× working live-dead stain solution. Samples were incubated at room temperature in the dark for 15 min and followed by readings on a Biotek Synergy 2 plate reader, where green and red fluorescence were measured with an excitation of 485 ± 20 nm and an emission of 528 ± 40 nm and 620 ± 40 nm, respectively.

### Bacterial cell wall protein collection

Bacteria were grown to mid-exponential phase OD_600_ ~0.6, normalized, and then fractionated for cell wall and released proteins as previously described (17). Briefly, bacterial cultures were centrifuged for 15 min at 12°C and 8,000 rpm. To precipitate released proteins, supernatants were recovered, and trichloroacetic acid (TCA) was added to a final concentration of 10%. The TCA-treated supernatants were incubated on ice for 1 h, and precipitated proteins were recovered by centrifugation for 15 min at 12°C and 8,000 rpm. The supernatants were discarded, and proteins were washed with ice-cold acetone, and pellets were collected by centrifugation, allowed to air dry, and then resuspended in STM buffer (0.5 M sucrose, 50 mM Tris-HCl at pH 8, 10 mM MgCl_2_, 2 µL protease inhibitor cocktail 3 [PIC3], and 3 mM NaAzide]. For cell wall proteins, cell pellets were washed with buffer containing 50 mM Tris-HCl at pH 8 and 10 mM MgCl_2_, then resuspended in STM buffer containing 100 mg/mL lysozyme and 20 µg/mL endolysin, and incubated for 15 min at 37°C; cell protoplast formation was monitored by microscopy; supernatants containing cell wall proteins were collected by centrifugation for 3 min at 12°C and 15,000 × *g*. Cell fractions were probed with antibodies specific for PrsA2 and InlA and previously published (17).

### Tandem mass tagged-mass spectrometry

TMT-MS was performed at the University of Illinois Chicago (UIC) Mass Spectrometry Facility as previously described (17), where filtered aided sample preparation was applied to extract and digest cellular proteins from all samples. In brief, 50 µg of each protein sample was dissolved in 8 M urea in 0.1 M Tris-HCl at pH 8.5 and then filtered with a 0.22 µM membrane. As indicated by the manufacturer, the flow-through was collected and transferred into a 1.5 mL Microcon YM-10 centrifugal unit (Millipore). Protein reduction, alkylation, and tryptic digestion were performed stepwise in the centrifugal unit as indicated by the manufacturer (Millipore). After overnight digestion at 37°C, the peptides were eluted twice with 50 µL 0.1% formic acid. The concentration of proteins and peptides collected in each step was measured using a Nanodrop ONE (Thermo Scientific). The digested peptides were then desalted, dried, and stored at −80°C until further use. Isobaric labeling was performed according to the manufacturer using 100 µg of peptides per sample for 10-plex TMT (Thermo Scientific). Excess reagents and detergents were removed by reversed phase solid phase extraction (Oasis HLB C18 SPE, Waters Corp.) after labeling. The samples were dried, and the resulting pellets were stored in − 80°C until further processing. High pH reversed-phase chromatography using Agilent 1260 HPLC and Waters XBridge column (c18 4.6 × 150 mm, 3.5 µm) was used to further fractionate samples. Ninety fractions were collected and combined into 10 fractions, followed by desalting using Nestgroup c18 tips. Fractionated peptides were dried and dissolved in 0.1% formic acid for liquid chromatography tandem mass spectrometry (LC-MS/MS) analysis. Over a 60-min gradient, fractions were run on a Thermo Fisher Orbitrap Velos Pro coupled with an Agilent NanoLC system. Samples were analyzed with a 60-min linear gradient (0%–35% acetonitrile with 0.1% formic acid), and data were acquired in a data-dependent manner, in which MS/MS fragmentation is performed on the top 12 intense peaks of every full MS scan. RAW files were converted into .mgf files using MSConvert (from ProteoWizard). Database search was carried out using the Mascot server (from Matrix Science). Search results from 18 runs were imported into Scaffold (Proteome Software, Portland, OR) for quantitative analysis.

### TMT-MS analysis

TMT-MS analysis was performed using data from two independent replicates for the cell wall and released fraction of each strain. The raw data, provided in log_2_ form, were linearized and normalized based on the optical density and control protein concentration of the wild-type sample. Normalized values in each experimental condition were divided by the corresponding normalized values in the wild-type control to calculate the fold change of proteins in each experimental condition. The fold change values for each protein in each experimental condition were then averaged. A student’s two-tailed t-test was performed on the normalized data of wild-type and experimental conditions to determine statistical significance (*P* ≤ 0.05).

### R analysis of TMT-MS data generation of Pearson’s correlation

Output data from the Scaffold of cell wall and released fractions were evaluated using the R package “DEP” (Differential Enrichment analysis of Proteomics data) (107). As per the package vignette, the variance stabilizing transformation (vsn) was used to normalize the data. All possible contrasts were used for differential enrichment analysis. Significant proteins were determined by alpha (in lieu of “*P*-value”) <0.05 and log fold change >log_2_ (0.5). Only proteins reaching these thresholds were included for purposes of downstream data visualization.

### Meta-analysis of gram-positive *prsA* homolog deletion mutant quantitative proteomic secretome data

To identify high-quality data sets, PubMed database searches were performed using the following key terms: “PrsA,” “parvulin,” “chaperone,” “secretion,” “proteome,” “secretome,” “Tandem Mass Spectrometry,” and “gram-positive,” combined with Boolean operators AND or OR. The references of the resulting publications were also assessed to identify additional sources. Tandem Mass Spectrometry data sets from selected publications were downloaded, and proteins with statistically significant alterations in secretion abundance in *prsA* homolog deletion strains relative to their respective wild type were identified. Statistical significance was defined as a Mascot score ≥ 70 in the *S. aureus* data set (23), logarithmic *t*-test *P* ≤ 0.05 for the *S. pyogenes* data set (25), and student’s two-tailed *t*-test *P* ≤ 0.05 for the *S. pneumoniae* (24) and *L. monocytogenes* data sets (17). UniProt and PubMed database searches were performed to determine the function and potential homologs of the identified proteins.

### Growth of *L. monocytogenes* under thermal, osmotic, and thermo-osmotic stress

*L. monocytogenes* was inoculated 1:20 in either plain BHI broth (Oxoid CM1135B) or BHI with 5% NaCl in sterile 125-mL flasks from saturated overnight cultures grown at 37°C, shaking at 180 rpm. Strains were cultured at 30°C, 37°C, and 42°C and measured at OD_600_ every hour for 8 h with a final reading at 24 h post-inoculation. Strains cultured at 16°C were measured in 8 and 24-h increments for 48 h. All strains were cultured shaking at 180 rpm for each designated temperature.

### Growth of *L. monocytogenes* under oxidative stress

*L. monocytogenes* strains were inoculated 1:20 from saturated overnight cultures into BHI broth and grown at 37°C and 180 rpm to OD_600_ ~0.6. Strains were then serially diluted in sterile ddH_2_O and spot plated onto BHI agar with 0.75 mM copper (II) chloride, 0.025 mM cadmium chloride, 4 mM hydrogen peroxide, or 1.25 mM diamide and grown overnight at 37°C. Colonies were enumerated, and CFUs per mL were calculated based on volume and dilution plated.

### Microscopy and quantification of cell length

*L. monocytogenes* strains were inoculated 1:20 from saturated overnight cultures into BHI broth and grown at 37°C and 180 rpm to OD_600_ ~ 0.6 or OD_600_ ~ 1.2, then normalized. On glass slides, 5 µL of each bacterial sample was pipetted in duplicate, covered with a glass coverslip, and sealed. Phase contrast microscopy was then performed with a 100× oil objective. Images were processed and analyzed using ImageJ (92) to quantify cell length.

To stain the bacterial membrane, 5 µL of 100 µg/ml Nile red solution was added to 100 µL of bacterial cells and incubated for 20 min at 37°C. The cells were washed twice with PBS and subsequently suspended in 50 µL of PBS. On glass slides, 5 µL of each bacterial sample was pipetted in duplicate, covered with a glass coverslip, and sealed. Phase contrast and fluorescence microscopy was then performed on an Olympus IX83 epifluorescence microscope using a 100× oil objective. Images were processed and analyzed using ImageJ (92).

### Statistical analyses

For data presented as bar graphs or violin plots, one-way ANOVA tests and Dunnett’s multiple comparison tests, with a single pooled variance, were used for statistical analysis, where *P* ≤ 0.05, and error bars represent the standard error of the mean. For live-dead XY scatter plots, to compare the slope and intercept of the linear regression lines, an F-test was used for statistical analysis. For XY line graphs, repeated measures two-way ANOVA tests and Dunnett’s multiple comparison tests, with a single pooled variance, were used where *P* ≤ 0.05, and error bars represent the standard error of the mean.

## EDITOR'S NOTE

**Ed. Note**: A potential conflict of interest was identified after acceptance of this paper, and the editor-in-chief performed an additional final review of the paper.

## SUPPLEMENTAL MATERIAL

Supplemental figures and tables jb.00531-25-s0001.pdf Figures S1 to S8, and Tables S4 and S5.

Table S1 jb.00531-25-s0002.xlsx TMT-MS analysis of all significantly altered protein profiles for *prsA* mutants.

Table S2 jb.00531-25-s0003.xlsx Proteins with statistically significant altered secretion profiles as measered by TMT-MS

Table S3 jb.00531-25-s0004.xlsx Proteins with Significantly Altered Secretion in Released Fractions of All *prsA2* mutants

ASM does not own the copyrights to Supplemental Material that may be linked to, or accessed through, an article. The authors have granted ASM a non-exclusive, world-wide license to publish the Supplemental Material files. Please contact the corresponding author directly for reuse.
