# Supplementary material for: The roles of the Listeria monocytogenes post-translocation chaperones PrsA1 and PrsA2 in protein secretion and stress resistance
Source: J Bacteriol. 2025 Dec 29;208(1):e00531-25. doi: 10.1128/jb.00531-25 (PMC12826049; doi:10.1128/jb.00531-25)
Supplement: Supplemental figures and tables — Figures S1 to S8, and Tables S4 and S5. [file jb.00531-25-s0001.pdf]

Supporting Information

The roles of the *Listeria monocytogenes* post-translocation chaperones PrsA1 and PrsA2 in protein secretion and stress resistance

Jada L. George[1], Leah F. Cabo[1], Jon P. Boyle[1], Nancy E. Freitag[2&3], Laty A. Cahoon[1]*

[1]Department of Biological Sciences, University of Pittsburgh, Pittsburgh, PA, USA
[2]Department of Microbiology and Immunology, University of Illinois at Chicago, Chicago, IL, USA
[3]Department of Pharmaceutical Sciences, University of Illinois at Chicago, Chicago, IL, USA

*Corresponding author: latycahoon@pitt.edu

Supplemental Figures
S1: TMT-MS analysis of all significantly altered protein profiles for *prsA* mutants.
S2: Bacterial viability and correlation analysis.
S3: Bacterial growth in minimal media.
S4: *L. monocytogenes* PrsA mutant growth at 16°C.
S5: Bacterial growth at 16 ºC BHI with 5% NaCl.
S6: Phase contrast microscopy of *L. monocytogenes prsA* mutants.
S7: The contribution of *L. monocytogenes prsA2* to cell length during mid-exponential phase.
S8: Trends in *L. monocytogenes prsA* mutant cell length and area at distinct growth phases.

Supplemental Tables
S1: TMT-MS analysis of *L. monocytogenes* PrsA secretion chaperone mutants (separate file)
S2: Significantly altered secreted proteins (separate file)
S3: Proteins with significantly altered secretion in released fractions of all PrsA2 mutants (separate file)
S4: Gram-positive genome and predicted proteome sizes
S5: Bacterial strains and designations

Figure S1

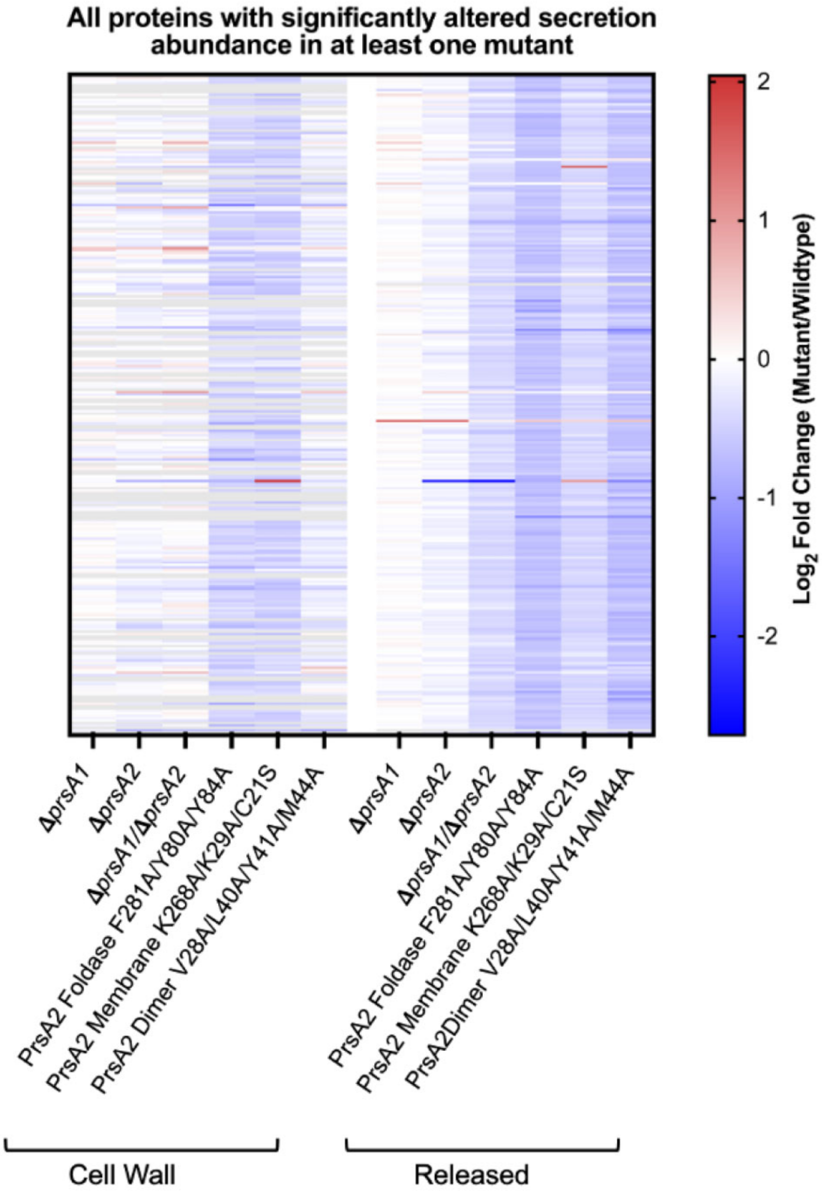

Figure S1. TMT-MS analysis of all significantly altered protein profiles for *prsA* mutants. Secretion profiles of Δ*prsA1* and Δ*prsA2* single and double mutants, and PrsA2 foldase domain, membrane interface, and dimer interface structural variant represented as a heat map. Relative amounts of *L. monocytogenes* mutant strain cell wall and released protein factors with significantly altered secretion levels in at least one strain as compared to wildtype are shown. The fold change (Log_2 of mutant/wildtype) are represented where negative fold change is dark blue, positive fold change is dark red, and no change is white. Proteins not detected in a fraction are in gray. Protein fold changes are depicted as an average of two independent experiments. Statistical significance determined as $P \leq 0.05$ by a student's two-tailed t-test.

Figure S2

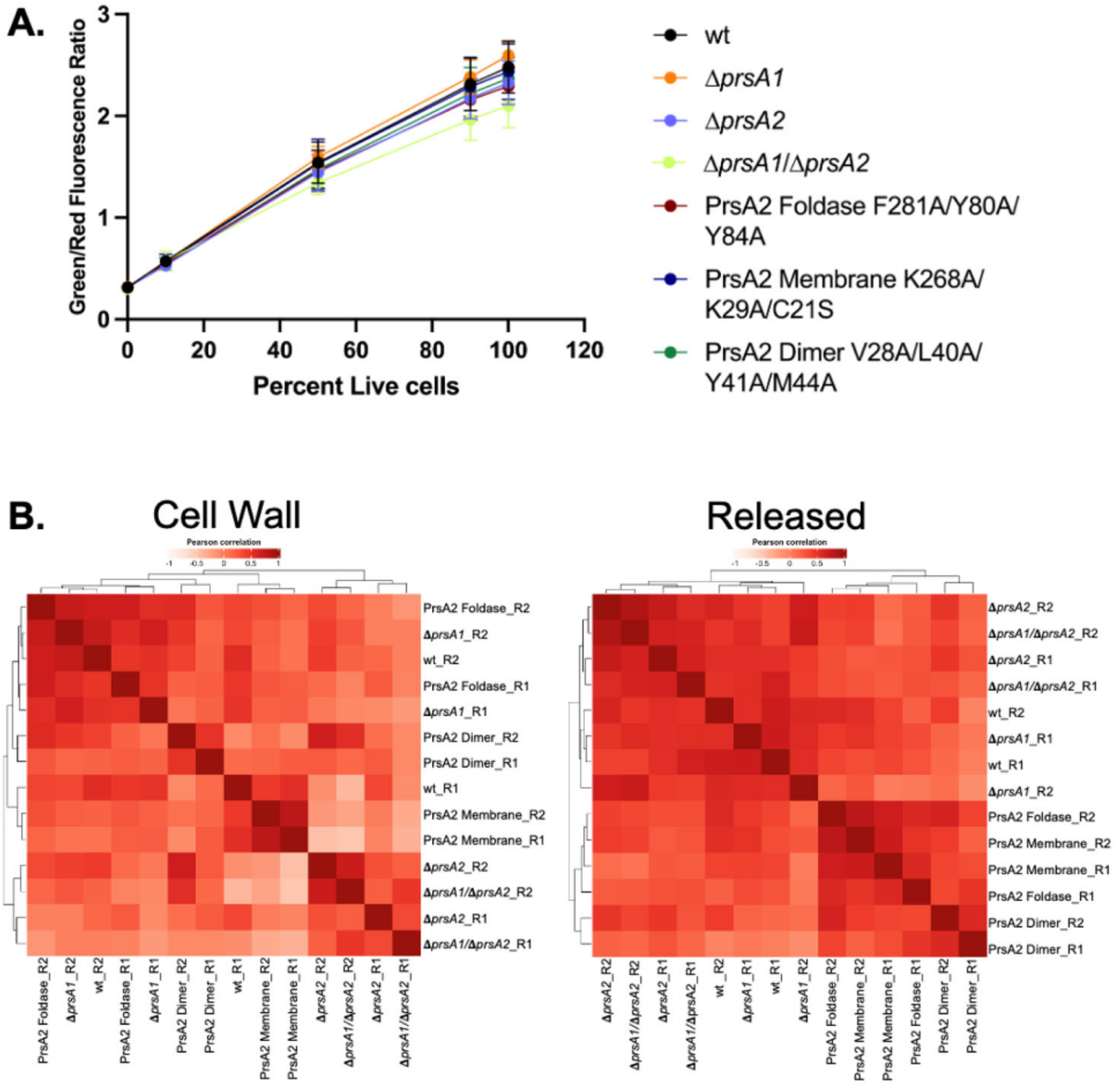

Figure S2. Bacterial viability and correlation analysis. (A) Relative viability of *L. monocytogenes prsA* strains measured by live-dead staining. The proportion of live-to-dead cells was calculated as the ratio of green-to-red fluorescence. Error bars represent the standard error of the mean of 4 independent experiments. The relationship between the ratio of the green-to-red fluorescence (y-axis) and the percent live bacteria (x-axis) is shown as a line using a linear least squares fit regression. All strains were statistically similar to wildtype as measured by F-test. (B) Heat maps demonstrating the Pearson's correlation between TMT-MS identified proteins from the cell wall (left) and released (right) fractions of *L. monocytogenes prsA* strains as evaluated by the R package "DEP" (Differential Enrichment analysis of Proteomics data)[1]. R1 and R2 represent replicate 1 and 2, respectively.

Figure S3

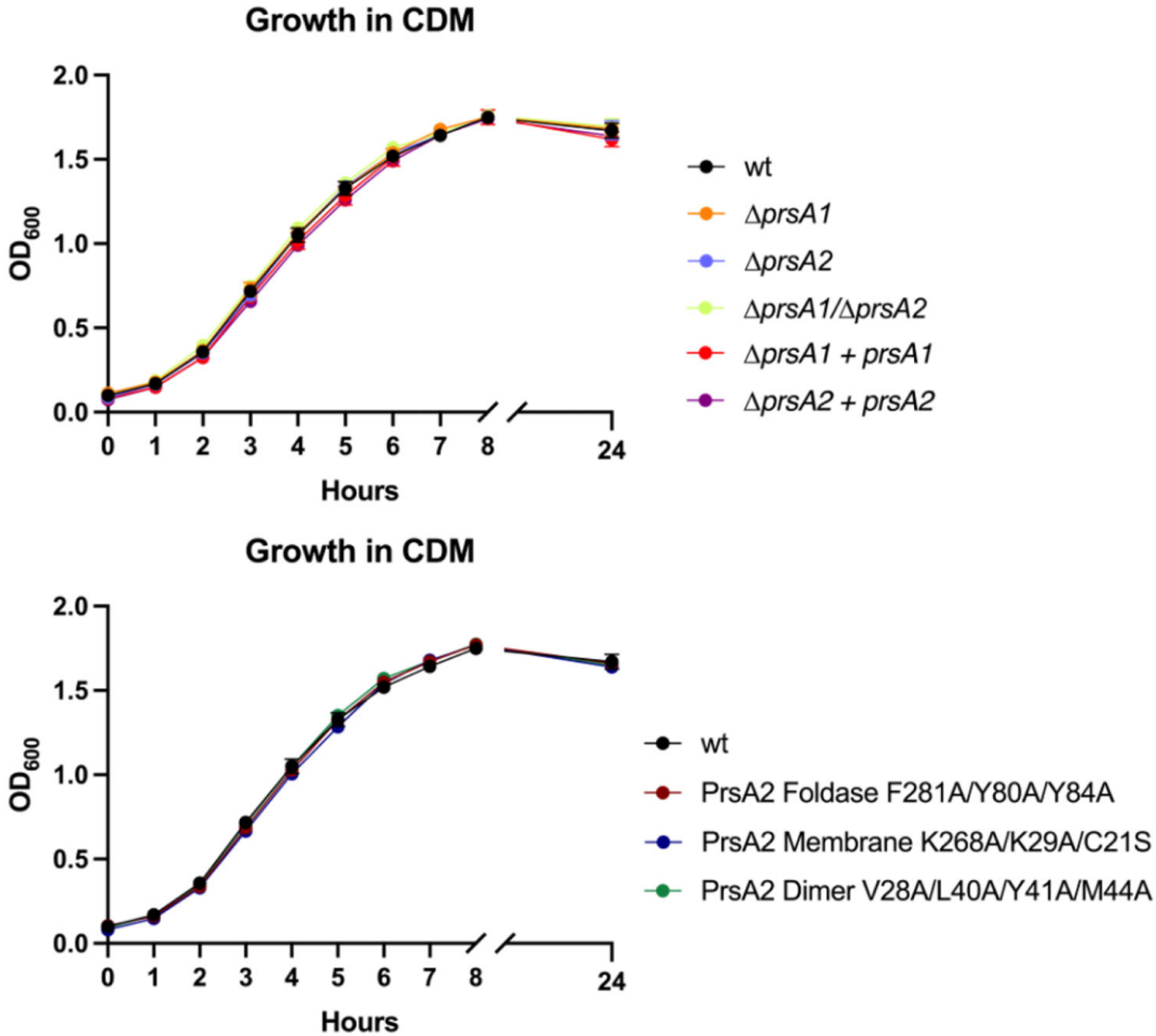

Figure S3. Bacterial growth in minimal media. Bacterial growth of the wildtype, Δ*prsA1*, Δ *prsA2,* Δ*prsA1*/Δ*prsA2,* foldase domain, membrane and dimer interface mutants and complement strains in minimal chemically defined media (CDM) over a period of 24 hours is shown. Error bars represent the standard error of the mean for 3 independent experiments. Asterisks indicate statistical significance of $P \leq 0.05$ by a repeated measures two-way ANOVA and Dunnett's multiple comparison test, with a single pooled variance.

Figure S4

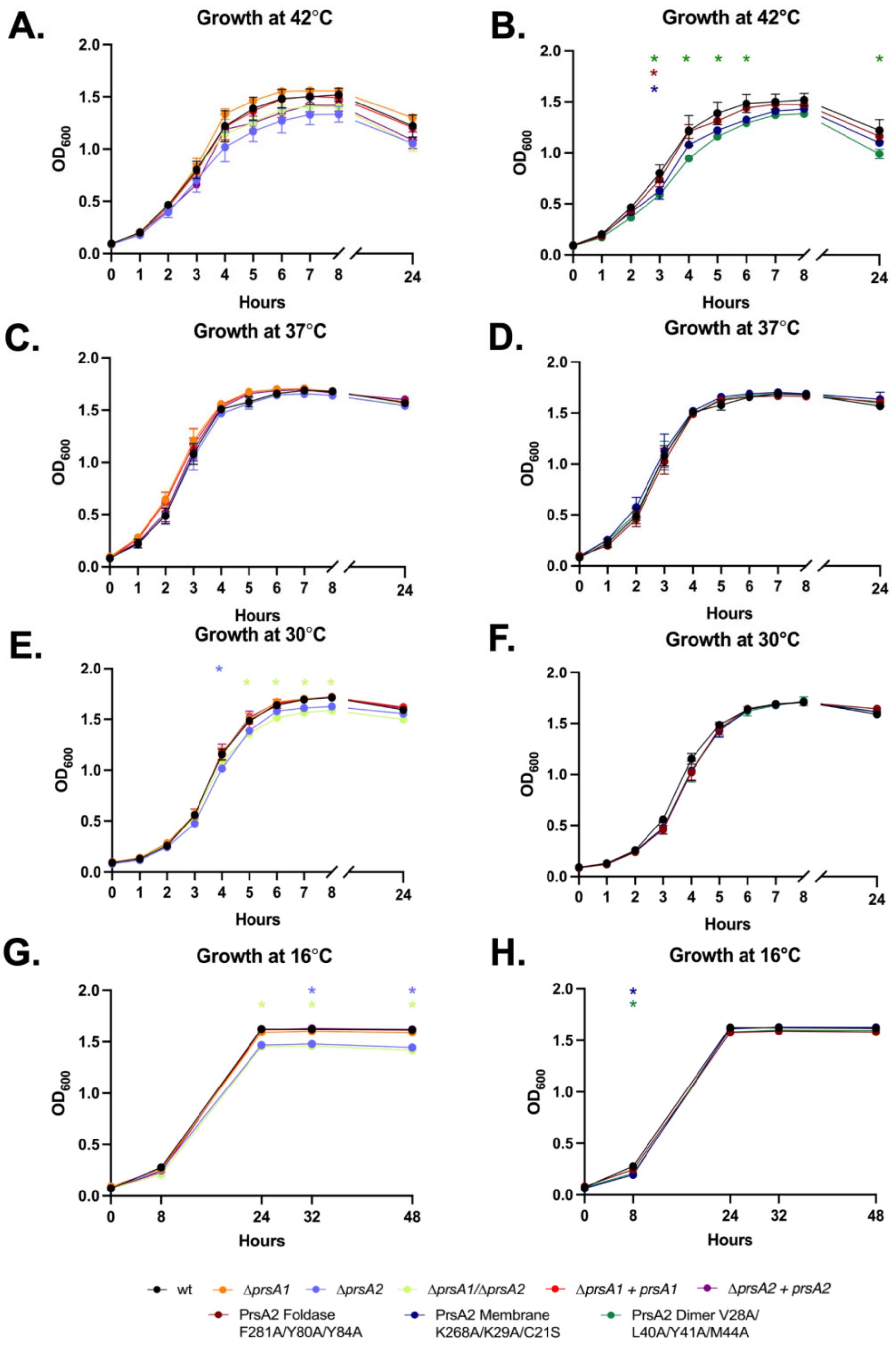

Figure S4. *L. monocytogenes* PrsA mutant growth at 16°C. Bacterial growth of the wildtype, *prsA2* and *prsA1* deletion mutants and complements (left) and foldase domain, dimer interface and membrane interface mutants (right) in BHI media. Optical density (OD) at 600nm was monitored over a period of 24 hours at 42°C (A & B), 37°C **(C & D)** and 30°C **(E & F)** or 48 hours at 16°C **(G & H)**. Error bars represent the standard error of the mean for 3 independent experiments. Asterisks indicate statistical significance of $P \leq 0.05$ by repeated measures two-way ANOVA tests and Dunnett's multiple comparison tests, with a single pooled variance.

Figure S5

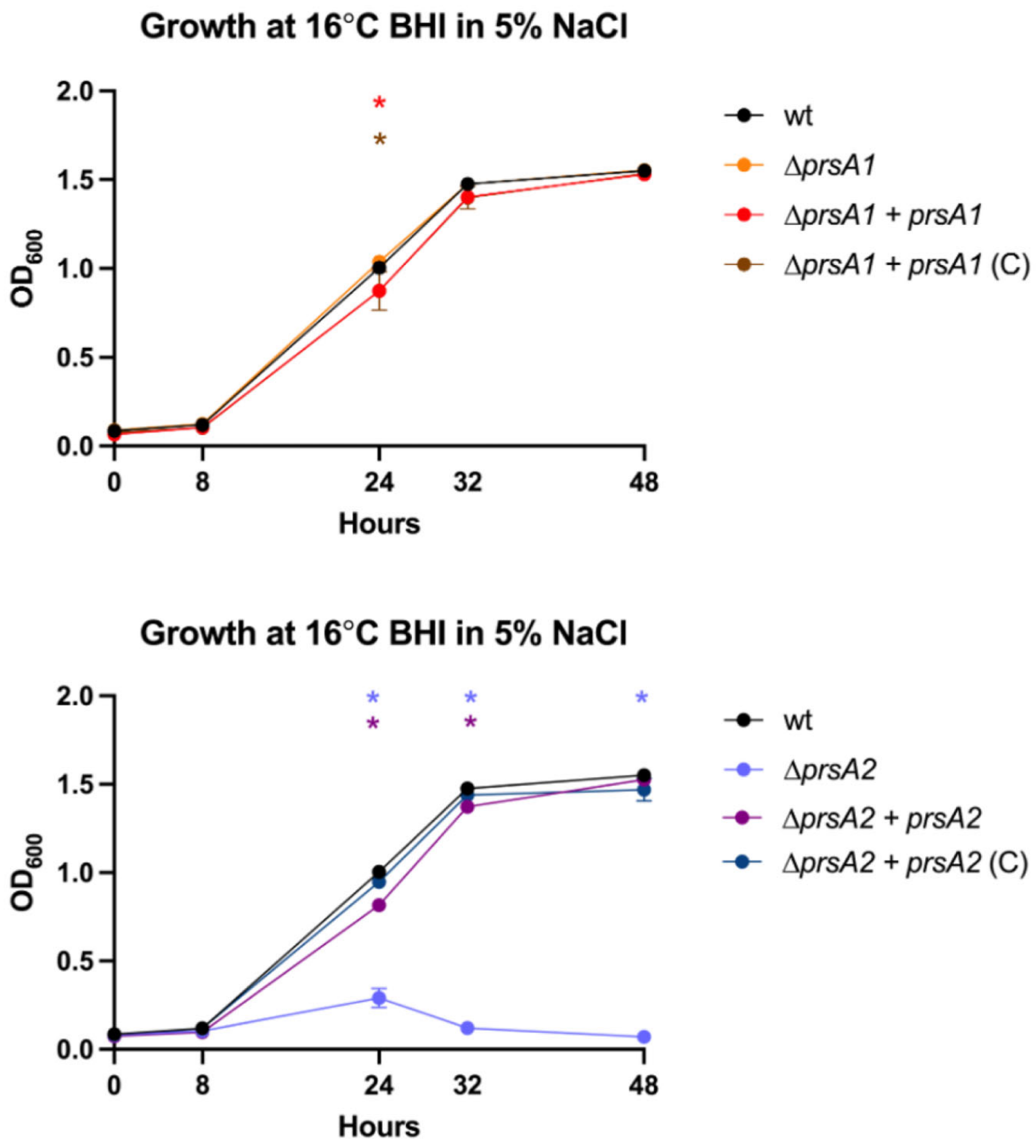

Figure S5. Bacterial growth at 16 ºC BHI with 5% NaCl. Bacterial growth of the wildtype, Δ*prsA1*, Δ*prsA2,* native promoter-inclusive complement strains, and constitutively active complement strains (C) over a period of 24 hours is shown. Error bars represent the standard error of the mean for 3 independent experiments. Asterisks indicate statistical significance of $P \leq 0.05$ by a repeated measures two-way ANOVA and Dunnett's multiple comparison test, with a single pooled variance.

Figure S6

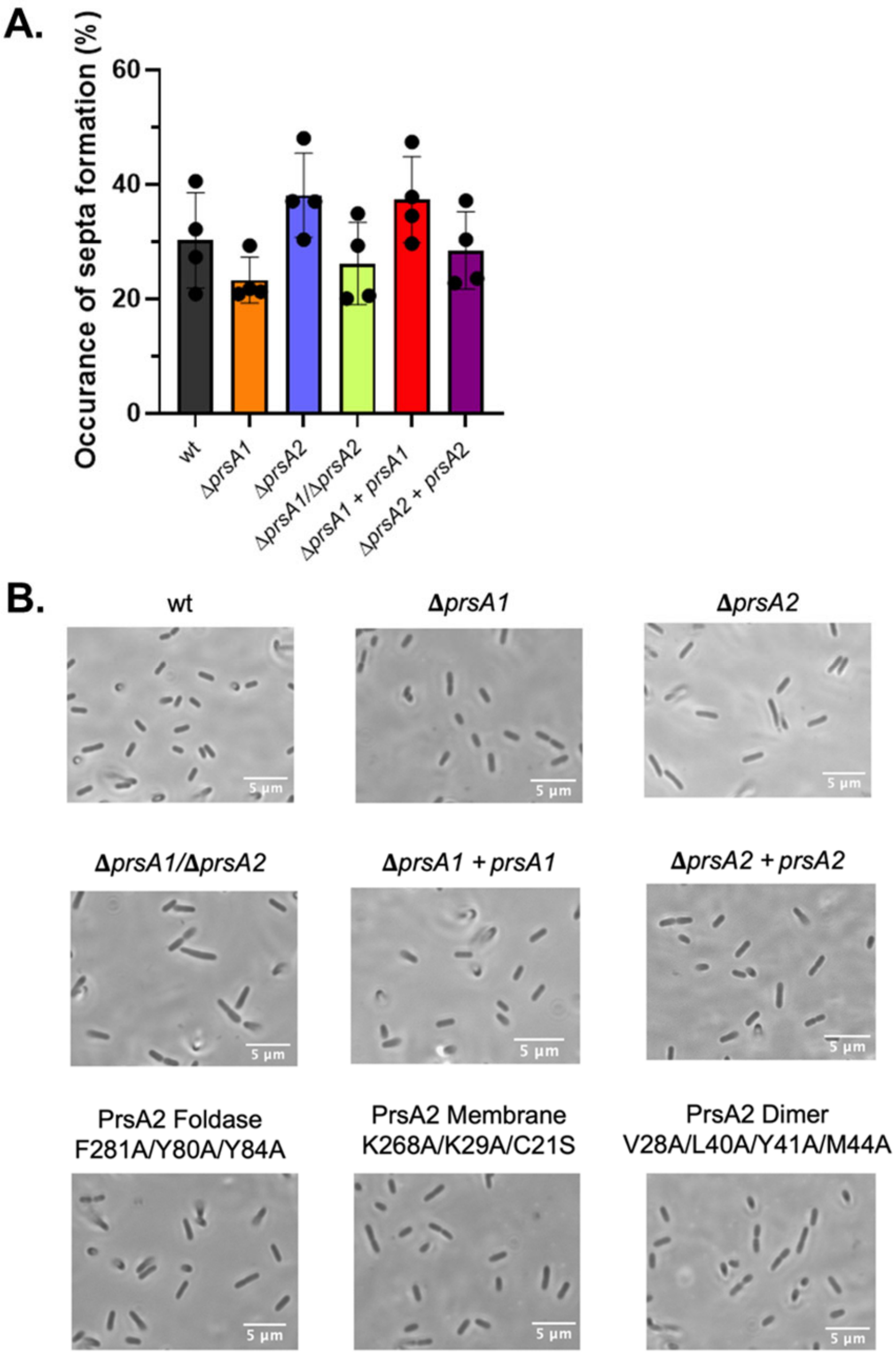

Figure S6. Phase contrast microscopy of *L. monocytogenes prsA* mutants. **(A)** Occurrence of septa formation in wildtype and *prsA* mutants at $OD_{600} \sim 1.2$, four independent microscopy images of Nile red stained-cells were measure totaling over 100 cells per indicated strain. No statistical significance was observed by Ordinary one-way ANOVA and Dunnett's multiple comparison test with a single pooled variance. Error bars represent the standard error of the mean. **(B)** Representative phase contrast microscopy images of wildtype and *prsA* mutants at $OD_{600} \sim 1.2$. Each strain was analyzed in duplicate. Images were taken with a 100X oil objective and scale bars represent 5 μm.

Figure S7

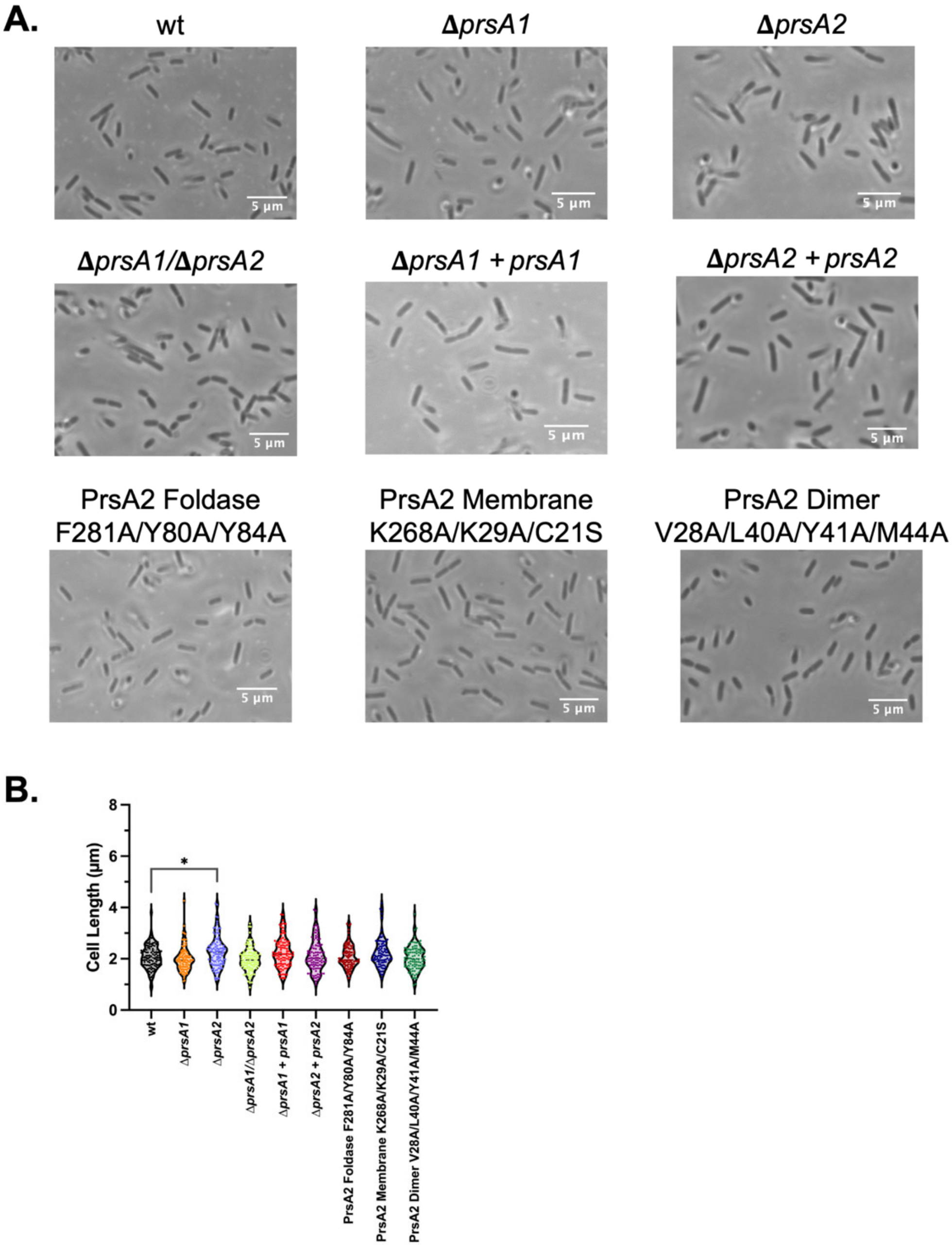

Figure S7. The contribution of *L. monocytogenes prsA2* to cell length during mid-exponential phase. **(A)** Representative phase contrast microscopy images of wildtype and *prsA* mutants at OD_{600} ~0.6. **(B)** Violin plots depicting the quantification of phase contrast microscopy analysis of the average length of *L. monocytogenes* cells. Phase contrast microscopy analysis was performed in duplicate for all samples, N = 100 cells for all samples. Cell length was quantified using ImageJ [2]. Asterisks indicate statistical significance of $P \leq 0.05$ by Brown-Forsythe and Welch ANOVA tests, and Dunnett's T3 multiple comparisons test, with individual variances computed for each comparison. Error bars represent the standard error of the mean. Images were taken with a 100X oil objective and scale bars represent 5 µm.

Figure S8

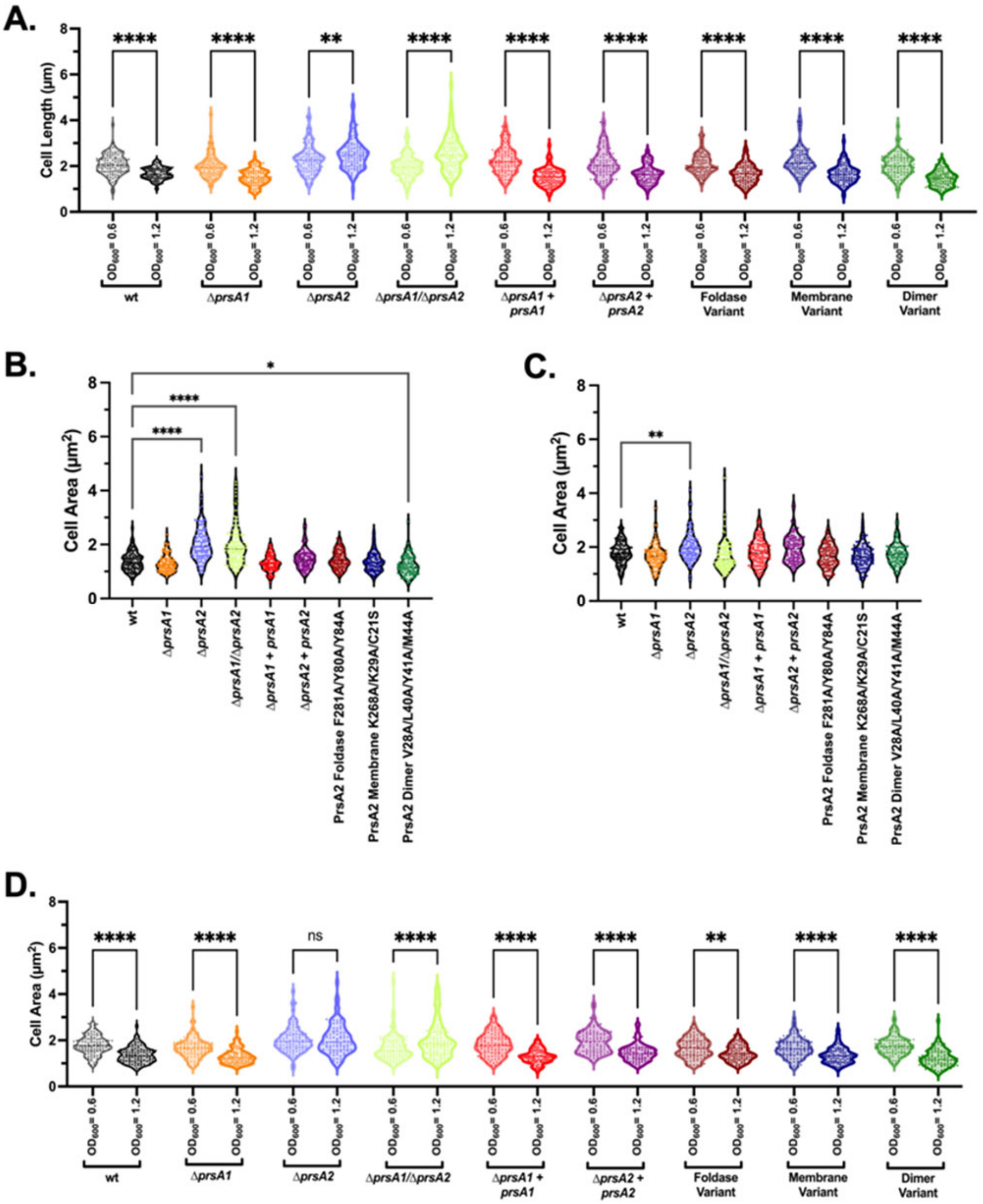

Figure S8. Trends in *L. monocytogenes prsA* mutant cell length and area at distinct growth phases. Violin plots depicting the trends in **(A)** cell length or (**D**) cell area during different growth phases and the quantification of phase contrast microscopy analysis of the average area of *L. monocytogenes* cells at **(B)** OD_{600} ~1.2 (early stationary phase) or at **(C)** OD_{600} ~0.6 (mid-exponential phase). Phase contrast microscopy analysis was performed in duplicate for all samples, N = 100 cells for all samples. Cell length was quantified using ImageJ [2]. Error bars represent the standard error of the mean. Asterisks indicate statistical significance of $P \leq 0.05$ by (**A** & **D**) Brown-Forsythe and Welch ANOVA tests, and Dunnett's T3 multiple comparisons test, with individual variances computed for each comparison or (**B** & **C**) Ordinary two-way ANOVA testes with main effects and Šídák's multiple comparisons test, with a single pooled variance.

Table S4. Gram-positive genome and predicted proteome sizes

	Genome size (kBps)	Predicted proteins
L. monocytogenes	2903	2844
S. pneumoniae	2161	2109
S. pyogenes	1852	1690
S. aureus	2821	2889

Table S4. The genome size as listed on GenBank and number of predicted proteins as determined by SignalP version 6.0 [3] analysis of the *L. monocytogenes* 10403S (Assembly: GCA_000168695.2, Taxon ID: 393133), *S. pneumoniae* TIGR4 (Assembly: GCA_000006885.1, Taxon ID: 170187), *S. pyogenes* M1 GAS (Assembly: GCA_000006785.2, Taxon ID: 160490), and *S. aureus* NCTC 8325 (Assembly: GCA_000013425.1, Taxon ID: 93061).

Table S5. Bacterial strains and designations

Strain or plasmid	Description	Designation	Reference
NF-L100	*L. monocytogenes* 10403S parent strain	wt	(Bishop & Hinrichs, 1987) [4]
NF-L1483	10403S with *prsA1* in-frame deletion	Δ*prsA1*	(Alonzo *et al.,* 2009) [5]
NF-L1651	10403S with Δ*prsA2*::*erm*	Δ*prsA2*	(Alonzo *et al.,* 2009) [5]
NF-L1631	NF-L1483 (Δ*prsA1*) transduced with Δ*prsA2*::*erm*	Δ*prsA2/*Δ*prsA1*	(Alonzo & Freitag, 2010) [6]
LAC-130	NF-L1483 (Δ*prsA1*) with integrated pPL2-*prsA1* wildtype (pNF-3860)	Δ*prsA1 + prsA1*	This work
NF-L3790	NF-L1651 with integrated pPL2-*prsA2* wildtype	Δ*prsA2 + prsA2*	(Cahoon *et al.,* 2016) [7]
NF-L3802	NF-L1651 with integrated pPL2-*prsA2* F281A/Y80A/Y84A	Foldase F281A/Y80A/Y84A	(Cahoon *et al.,* 2016) [7]
NF-L3796	NF-L1651 with integrated pPL2-*prsA2* K268A/K29A/C21S	Membrane K268A/K29A/C21S	(Cahoon *et al.,* 2016) [7]
NF-L3806	NF-L1651 with integrated pPL2-*prsA2* V28A/L40A/Y41A/M44A	Dimer V28A/L40A/Y41A/M44A	(Cahoon *et al.,* 2016) [7]
NF-L4075	NF-L1483 (Δ*prsA1*) with integrated pIMK2-*prsA1* (pNF-3664)	Δ*prsA1 + prsA1* (C)	(Cahoon *et al.,* 2022) [8]
NF-L3632	NF-L1651 with integrated pIMK2-*prsA2* (pNF-3662)	Δ*prsA2 + prsA2* (C)	(Cahoon & Freitag, 2015) [9]
pNF-3860	pPL2-*prsA1* (containing the open reading frame from *prsA1* including 195 bp upstream and 66 bp downstream region)		(Cahoon *et al.,* 2022) [8]
pNF-3664	pIMK2 with *prsA1*		(Cahoon & Freitag, 2015) [9]
pNF-3662	pIMK2 with *prsA2*		(Cahoon & Freitag, 2015) [9]

Supplemental References

1. Zhang, X., et al., *Proteome-wide identification of ubiquitin interactions using UbIA-MS.* Nat Protoc, 2018. **13**(3): p. 530-550.
2. Schneider, C.A., W.S. Rasband, and K.W. Eliceiri, *NIH Image to ImageJ: 25 years of image analysis.* Nat Methods, 2012. **9**(7): p. 671-5.
3. Teufel, F., et al., *SignalP 6.0 predicts all five types of signal peptides using protein language models.* Nat Biotechnol, 2022. **40**(7): p. 1023-1025.
4. Bishop, D.K. and D.J. Hinrichs, *Adoptive transfer of immunity to Listeria monocytogenes. The influence of in vitro stimulation on lymphocyte subset requirements.* J Immunol, 1987. **139**(6): p. 2005-9.
5. Alonzo, F., 3rd, et al., *The posttranslocation chaperone PrsA2 contributes to multiple facets of Listeria monocytogenes pathogenesis.* Infect Immun, 2009. **77**(7): p. 2612-23.
6. Alonzo, F., 3rd and N.E. Freitag, *Listeria monocytogenes PrsA2 is required for virulence factor secretion and bacterial viability within the host cell cytosol.* Infect Immun, 2010. **78**(11): p. 4944-57.
7. Cahoon, L.A., N.E. Freitag, and G. Prehna, *A structural comparison of Listeria monocytogenes protein chaperones PrsA1 and PrsA2 reveals molecular features required for virulence.* Mol Microbiol, 2016. **101**(1): p. 42-61.
8. Cahoon, L.A., et al., *Listeria monocytogenes two component system PieRS regulates secretion chaperones PrsA1 and PrsA2 and enhances bacterial translocation across the intestine.* Mol Microbiol, 2022. **118**(3): p. 278-293.
9. Cahoon, L.A. and N.E. Freitag, *Identification of Conserved and Species-Specific Functions of the Listeria monocytogenes PrsA2 Secretion Chaperone.* Infect Immun, 2015. **83**(10): p. 4028-41.
